# The oxidative aging model integrated various risk factors in type 2 diabetes mellitus at system level

**DOI:** 10.3389/fendo.2023.1196293

**Published:** 2023-05-24

**Authors:** Yao Chen, Lilin Yao, Shuheng Zhao, Mengchu Xu, Siwei Ren, Lu Xie, Lei Liu, Yin Wang

**Affiliations:** ^1^ Department of Biomedical Engineering, School of Intelligent Medicine, China Medical University, Shenyang, Liaoning, China; ^2^ Shanghai-MOST Key Laboratory of Health and Disease Genomics & Institute for Genome and Bioinformatics, Shanghai Institute for Biomedical and Pharmaceutical Technologies, Shanghai, China; ^3^ Intelligent Medicine Institute, Fudan University, Shanghai, China; ^4^ Key Laboratory of GI Cancer Etiology and Prevention in Liaoning Province, The First Hospital of China Medical University, Shenyang, China

**Keywords:** oxidative stress, type 2 diabetes mellitus, energy metabolism, aging, pan-cancer analysis

## Abstract

**Background:**

Type 2 diabetes mellitus (T2DM) is a chronic endocrine metabolic disease caused by insulin dysregulation. Studies have shown that aging-related oxidative stress (as “oxidative aging”) play a critical role in the onset and progression of T2DM, by leading to an energy metabolism imbalance. However, the precise mechanisms through which oxidative aging lead to T2DM are yet to be fully comprehended. Thus, it is urgent to integrate the underlying mechanisms between oxidative aging and T2DM, where meaningful prediction models based on relative profiles are needed.

**Methods:**

First, machine learning was used to build the aging model and disease model. Next, an integrated oxidative aging model was employed to identify crucial oxidative aging risk factors. Finally, a series of bioinformatic analyses (including network, enrichment, sensitivity, and pan-cancer analyses) were used to explore potential mechanisms underlying oxidative aging and T2DM.

**Results:**

The study revealed a close relationship between oxidative aging and T2DM. Our results indicate that nutritional metabolism, inflammation response, mitochondrial function, and protein homeostasis are key factors involved in the interplay between oxidative aging and T2DM, even indicating key indices across different cancer types. Therefore, various risk factors in T2DM were integrated, and the theories of oxi-inflamm-aging and cellular senescence were also confirmed.

**Conclusion:**

In sum, our study successfully integrated the underlying mechanisms linking oxidative aging and T2DM through a series of computational methodologies.

## 1 Introduction

Type 2 diabetes mellitus (T2DM) is a chronic endocrine metabolic disease caused mostly by insulin dysfunction. The increasing prevalence of diabetes has resulted in a great economic burden in many countries (1). According to statistics, there are approximately 536.6 million people with diabetes worldwide, and this number is expected to rise to approximately 783.2 million in 2045, with T2DM accounting for approximately 90% (1, 2). Therefore, it is imperative to study the etiology of T2DM in depth.

Various reports have shown that T2DM is closely related to aging, with aging being one of the most vital risk factors for T2DM (3, 4). Adipose tissue (AT) is redistributed during aging, which affects the sensitivity of insulin (5). Furthermore, the normal function of pancreatic beta cells also declines (3), and aging causes inflammation and low nutritional status, affecting the endocrine system (6). Additionally, a series of risk factors for T2DM are vital to other age-related diseases, such as Alzheimer's disease (AD), cardiovascular disease (CVD), and cancer (7–10).

**Figure 1:**
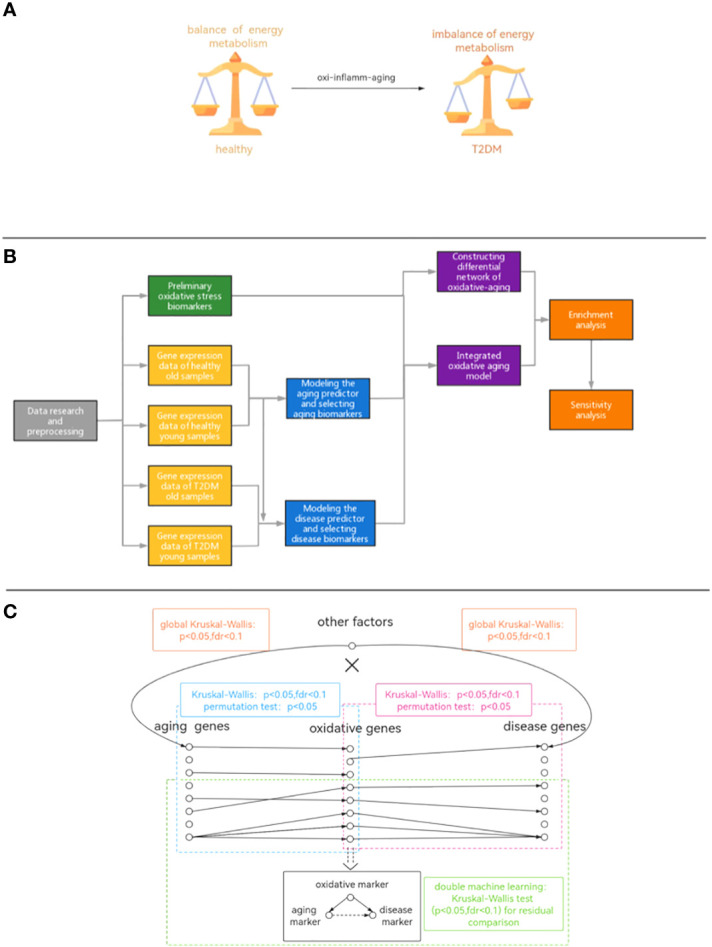
**(A)** Diagram of the hypothetical mechanism. **(B)** The workflow of our study. **(C)** The pipeline of integrated oxidative aging model.

During the aging process, oxidative stress accumulates, leading to an energy imbalance that is key to T2DM (11, 12). For example, oxidative intermediates can damage pancreatic beta cells and exacerbate insulin resistance (13). Moreover, accumulated reactive oxygen species also accelerate aging-related DNA damage and induce cellular senescence (14, 15). With increasing age, the free radical dynamic balance in cells is gradually broken, causing an increase in free radical concentration and inducing the oxidation reaction, leading to T2DM (16). In addition, oxidative stress is closely interrelated with inflammation (17) by activating multiple transcription factors in the inflammatory response (18). Furthermore, abnormal oxidative stress dysregulates the balance of energy metabolism during T2DM development (19–23). In summary, the potential mechanism by which aging-related oxidative stress (often described as “oxidative aging” (24)) triggers T2DM needs to be further studied at the system level (**Figure 1A**).

With the development of artificial intelligence, many research results on diabetes have utilized machine learning (ML), which can gain useful information from original profiles. ML can be widely used in the risk prediction, prognosis, and treatment of clinical diseases such as cardiovascular disease and cancer (25, 26). Recently, it was reported that ML can predict the occurrence of T2DM and its complications, as well as identify key markers in T2DM (27–29). Additionally, Mendelian randomization (MR) is conducive to integrating biological information (30, 31). Although numerous studies have revealed some risk factors/mechanisms associated with T2DM, the underlying mechanism between oxidative aging and T2DM is still unclear and requires further exploration.

To further explore the potential mechanisms between oxidative aging and T2DM, a series of computational studies was performed in this paper (**Figures 1B, C**): (1) Machine learning was used to identify aging and disease (T2DM) markers. (2) An integrative model was built to further explore essential relationships between oxidative aging (aging-related oxidative stress) and T2DM (**Figure 1C**). (3) Network analysis, enrichment analysis and sensitivity analysis were used to investigate the underlying mechanisms between oxidative aging and T2DM markers. (4) Relative biological functions of identified oxidative aging markers were further validated across different cancer types. As a result, the underlying mechanisms of T2DM (i.e., nutritional metabolism, inflammatory response, mitochondrial function and protein homeostasis) were integrated, which can also provide key indices in cancers.

## Results

### 2.1 Modeling prediction models and identifying relative biomarkers

**Figure 2:**
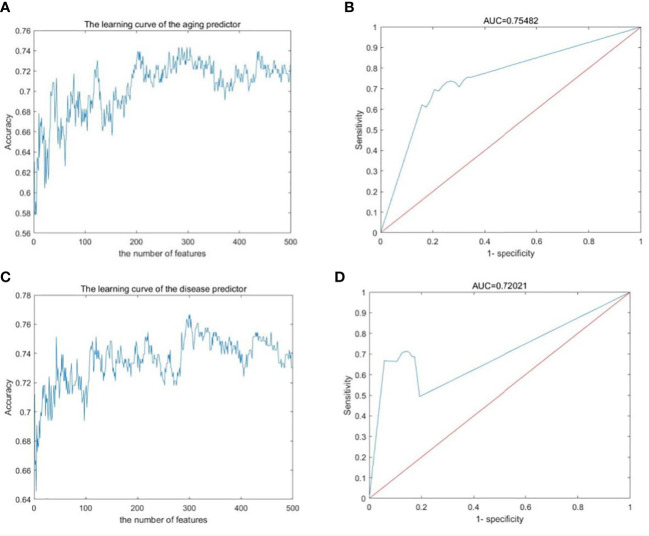
Machine learning results. **(A, B)** Aging predictor from our previous study, selecting the number of aging markers. **(C, D)** The improved inflamm-aging predictor, selecting the number of disease markers. **(A, C)** Learning curve for the training dataset. **(B, D)** The ROC curve for the test dataset.

The gene expression profiles were obtained from the GEO database, including 489 samples and 12,958 genes (**Tables S1**–**S3**). These genes were ranked by the ReliefF algorithm, and then the aging predictor and disease predictor were built using the k-nearest neighbors (kNN; k=3 with the correlation distance) algorithm, optimized by 10-fold cross-validation. The accuracy of the aging predictor in the test set was 0.70455 and 0.7279 in the aging and disease predictors (**Figure 1**; **Table 1**), respectively. Furthermore, the ROC area under the curve (AUC) for the aging and disease predictor models were 0.7712 and 0.72788 (**Figure 2**), respectively. As a result, our predictors were sufficiently accurate in both aging and disease models.

**Table 1:** The accuracy of aging predictor and disease predictor.

	The accuracy of training datasets	The accuracy of test datasets	Markers used for classification
The aging model	0.7552	0.70455	304
The disease predictor	0.8328	0.7279	299
The integrated oxidative aging model	0.8485	0.7662	282

Both aging and disease markers have meaningful biological functions. For example, OSBPL1A (oxysterol binding protein-like 1A, ReliefF weight=0.058) was the top aging marker. OSBPL1A is one of a set of intracellular lipid receptors and is closely related to lipid metabolism and cholesterol metabolism (32, 33). TIGD4 (tigger transposable element derived 4, ReliefF weight=0.0253), as the top disease marker, was related to glycogen metabolism. In sum, the abnormal metabolism of lipids, cholesterol and glycogen can lead to T2DM (34). These results indicated the crucial role of energy metabolism in T2DM.

### 2.2 Identifying the oxidative-aging risk factors by the integrated prediction model

The integrated oxidative aging model was built to explore essential relationships among aging, oxidative and T2DM markers (details are shown in Materials and Methods 5.3, with a total of 11829 “aging-oxidative-disease” triples). The top 10 aging, oxidative and disease markers are shown in **Table 2**, including relative experimental details (35–43). For example, ADP-ribosylarginine hydrolase (ADPRH) is the top aging marker, participating in the regulation of various cellular processes, including both immunity and aging (44). ADPRH adversely influences the immune system via CD8+ T cells, hence promoting an imbalance in energy metabolism (45). TPST1 (tyrosyl protein sulfotransferase 1) is the top disease marker, catalyzing the posttranslational sulfation of tyrosine residues within acidic motifs of many polypeptides in all multicellular organisms (46). TPST1 promoted the secretion of some cytokines and then induced the inflammatory response (47). COX5A (cytochrome C oxidase subunit 5A) is the top oxidative marker related to mitochondrial function (48), which induces an imbalance in energy metabolism and insulin resistance (35). In addition, the predictor accuracy calculated by the selected disease markers was 0.7662 (**Table 1**). In sum, these results indicated that the integrated oxidative aging model could identify essential relationships in T2DM, even with enough prediction ability.

**Table 2:** The top 10 aging markers, disease markers and oxidative markers from the integrated oxidative model.

Aging marker	Times	Disease marker	Times	Oxidative marker	Times	Experimental results of the oxidative marker	Reference	Experimental method
ADPRH	21	TPST1	26	COX5A	86	COX5A is related to mitochondrial dysfunction in insulin resistance.	(35)	Western blotting
OAS3	14	PGK1	25	CYB5B	83	CYB5B is related to diabetic retinopathy.	(36)	Quantitative PCR
RNF10	13	ADM	25	ERCC8	77	Loss of ERCC8 will have insulin-dependent diabetes with Cockayne syndrome.	(37)	DNA hybridization
LMO7	11	PLAC8	24	ANXA1	62	ANXA1 is related to weight gain and diet-induced insulin resistance.	(38)	Flow cytometry
KATNB1	10	ITGB5	22	ATRN	62			
PLD1	10	STEAP4	21	BAK1	59	BAK1 is related to mitochondria-dependent programmed cell death.	(39)	Cell culture of hepathocellular carcinoma and renal epithelial
PTPLB	9	TMEM163	21	CD36	58	CD36 is a key molecule to limit β-cell function in T2DM associated with obesity.	(40)	Western blot analysis
ATP1B3	9	KDELR3	20	CYCS	55	CYCS affects the expression level of β cells through regulating the production of mitochondrial ROS.	(41)	Western blot analysis
PABPC3	7	SCD	19	ALOX5	53	ALOX5 can lead to inflammation in patients with T2DM.	(42)	Normal fasting glucose and normal glucose tolerance
AQR	7	PELO	19	CAT	53	CAT belongs to peroxidase, which can affect the oxidative metabolism of fatty acid.	(43)	Cell culture of human fibroblasts

### 2.3 Sensitivity analysis further highlighted the imbalance of energy metabolism in T2DM

The Markov chain Monte Carlo (MCMC) method was used to evaluate the sensitive relationship between oxidative aging and T2DM. As a result, a series of triples were identified as key components (2501 out of 11829) in the integrated oxidative aging model.

The top 10 sensitive relationships (by calculating the absolute differential frequency) are shown in **Table 3**, where the top relationship was “OSBPL7-COX7C-TM6SF1” (difference=-0.03935). Additionally, **Table 3** also displayed experimental details of relative oxidative markers (49–55). OSBPL7 (oxysterol binding protein like 7) is an oxysterol-binding protein-like (OSBPL) family member involved in lipid binding and transport and induces cholesterol efflux (56, 57). COX7C (cytochrome C oxidase subunit 7C) is an enzyme in the electron transport chain related to cellular respiration and is also a potential biomarker of diabetes mellitus (58, 59). Transmembrane 6 superfamily member 1 (TM6SF1) participates in regulating transmembrane transport in macrophages (60). Overall, these results indicated that oxidative stress played an important role in the development of T2DM.

**Table 3:** The top 10 pairs with the greatest absolute difference frequency.

Aging marker	Oxidative marker	Disease marker	Difference	Experimental results of the oxidative marker	Reference	Experimental method
OSBPL7	COX7C	TM6SF1	-0.039347869	COX7C activity is associated with pancreatic β-cells.	(49)	OGTT testing
DNAJA3	MYC	GSTZ1	-0.037033525	MYC is a key factor for proliferation of pancreatic β-cells.	(50)	Western blot analysis and real-time PCR
OSBPL7	COX7C	SLC25A37	-0.036323552	COX7C activity is associated with pancreatic β-cells.	(49)	OGTT testing
OSBPL7	MGAT3	SF3A2	-0.0357659	MGAT3 plays role in lipid homeostasis.	(51)	Mouse model:oral administration of isoindoline-5-sulfonamide
TTC25	COX7A1	CMTM8	-0.03019756	COX7A1 activity is associated with pancreatic β-cells.	(49)	OGTT testing
OSBPL7	MGAT3	RECK	-0.027526704	MGAT3 plays role in lipid homeostasis.	(51)	Mouse model:oral administration of isoindoline-5-sulfonamide
MTUS1	ISCU	ATP5J	-0.019164871	ISCU can cause Friedreich ataxia (FRDA), which is related to diabetes.	(52)	Cell culture of endocardium
SLC23A2	GCH1	SPI1	0.01780268	GCH1 is related to endothelial dysfunction in T2DM.	(53)	Venous occlusion plethysmography
EPN1	IL18BP	MRPL11	0.017333862	IL18BP is related to inflammatory response, which plays important roles in diabetic nephropathy.	(54)	Cell culture of human proximal tubular epithelial and western blot analysis
EPN1	PARK7	NFKBIA	0.014743576	PARK7 participates in glucose homeostasis and then induces insulin resistance.	(55)	Quantitative PCR analysis and western blotting analyses

The top sensitive aging, disease, oxidative markers (evaluated by the occurrence times, also along with relative experimental details (61–69)) and are also shown in **Table 4**. For example, the top aging marker was HPS1 (Hermansky-Pudlak Syndrome 1 gene), inducing the biogenesis of lysosome-associated cellular organelles (70), which regulates the aging process through sphingolipids (71). The top disease marker was PPP1R15A (protein phosphatase 1 regulatory subunit 15A). PPP1R15A plays an important role in insulin resistance via energy metabolism (72, 73). The top oxidative marker was ATOX1 (antioxidant 1 copper chaperone). It has been reported that ATOX1 can regulate the copper level in the cell and maintain the redox balance as a defense antioxidant (74, 75). In short, the sensitivity analysis emphasized the crucial relationship among aging, oxidative stress and T2DM.

**Table 4:** The top 10 aging markers with the most paired with oxidative markers after sensitive analysis.

Aging marker	Times	Disease marker	Times	Oxidative marker	Times	Experimental results of the oxidative marker	Reference	Experimental method
HPS1	13	PPP1R15A	18	ATOX1	39	ATOX1 can protect pancreatic β-cells and induce diabetes mellitus.	(61)	Western blot analysis
SCARB1	10	ALDH4A1	16	APEX1	31	APEX1 is associated with diabetic retinopathy.	(62)	Western blot analysis
TMPO	7	G0S2	15	APP	29	APP is related to protein accumulation, and then leads to T2DM.	(63)	*In vitro* aggregation assay
MRPL10	7	CALML4	15	ALDH3B1	27	ALDH3B1 is related to lipid peroxidation.	(64)	Western blot analysis
TTC25	6	STXBP2	15	AXL	25	AXL is involved in diabetic vascular disease.	(65)	OGTT testing
MYLK	6	ZCCHC14	15	AKT1	24	AKT1 is related to insulin resistance.	(66)	Western blotting analysis and real-time PCR
RPS4Y1	5	MCEE	15	ARNTL	21	ARNTL regulates lipid metabolism and diet-induced insulin resistance.	(67)	Plasma metabolites analysis
ESCIT	5	HIST1H2AC	15	ADAM9	20	ADAM9 is a potential novel target for regulating the function of diabetic EPCs.	(68)	Western blotting
FKBP1B	5	PDLIM1	15	ATRN	20			
PTPLB	5	MRPL18	14	CAMKK2	20	CAMKK2 plays role in diet-induced obesity, glucose intolerance and insulin resistance.	(69)	Immunoblotting

### 2.4 Underlying oxidative-aging mechanisms based on enrichment analysis

**Figure 3:**
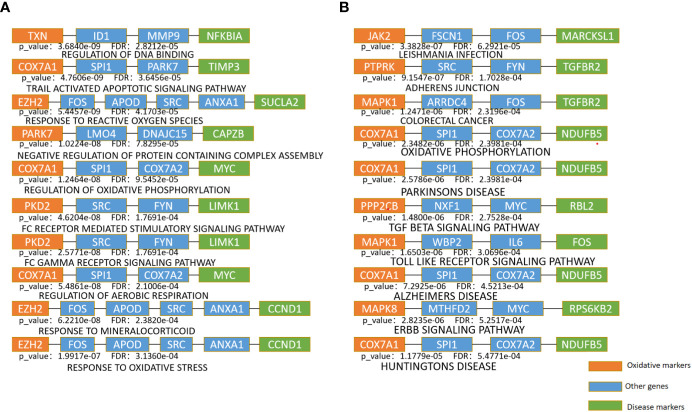
Enrichment analysis of the shortest path of KEGG and BP **(A)**. The top 10 pathway enrichment with the minimum FDR in BP terms **(B)**. The top 10 pathway enrichment with the minimum FDR in KEGG pathways.

To further explore the underlying mechanisms between oxidative aging and T2DM, the shortest path between each pair of oxidative aging and disease markers was identified, and then enrichment analysis was performed based on the Kyoto Encyclopedia of Genes and Genomes (KEGG) pathway and biological process (BP) terms in Gene Ontology (GO). As a result, relative enrichment results were summarized in **Figures 3**, **S1**, as well as **Tables 5** (75–88), **6** (89–102) and **S4** (76–81, 83–85, 103, 104, 111, 113), **S5** (89–91, 94, 96, 103, 105–110).

**Table 5:** The top 10 enriched KEGG pathways.

KEGG	Enriched shortest paths	Functions	Reference
PARKINSON DISEASE	1213	(1) T2DM and Parkinson Disease have shared pathological mechanism. (2) T2DM is a determinant of Parkinson Disease risk and progression.	(76–78)
OXIDATIVE PHOSPHORYLATION	1175	Causing metabolic alterations at the organism level through producing energy-rich molecules like ATP.	(87)
ALZHEIMERS DISEASE	1128	(1) T2DM is modifiable risk factor for Alzheimer’s Disease. (2) Insulin resistance is a common mechanism between Alzheimer’s Disease and T2DM.	(79, 80)
HUNTINGTONS DISEASE	1115	T2DM and Huntington’s Disease have shared treatment method.	(81)
LEISHMANIA INFECTION	702	Related to the immune system.	(82)
CARDIAC MUSCLE CONTRACTION	357	Related to insulin sensitivity and mitochondrial function.	(88)
TOLL LIKE RECEPTOR SIGNALING PATHWAY	239	Producing and releasing various inflammatory mediators and triggering immune response.	(83)
COLORECTAL CANCER	163	T2DM is the risk factor for colorectal cancer.	(84)
ADHERENS JUNCTION	145	Regulating insulin vesicle trafficking.	(85)
T CELL RECEPTOR SIGNALING PATHWAY	84	Related to immune system.	(86)

**Table 6:** The top 10 enriched BP terms.

BP	Enriched shortest paths	Functions	Reference
RESPONSE TO REACTIVE OXYGEN SPECIES	418	(1) Modifying cell signaling proteins and then mediating T2DM. (2) As a central mechanism for the development of T2DM.	(89, 101)
RESPONSE TO OXIDATIVE STRESS	308	(1) Causing the function of pancreatic beta cells damaged. (2) Related to insulin resistance.	(90, 91)
CELLULAR RESPONSE TO REACTIVE OXYGEN SPECIES	260	(1) Maintaining the cellular redox homeostasis. (2) Related to mitochondrial oxidative stress and cell senescence.	(92, 93)
CELLULAR RESPONSE TO CHEMICAL STRESS	179	Regulating the cellular redox state.	(94)
RESPONSE TO OXYGEN CONTAINING COMPOUND	142	Controlling the intracellular metabolism and energy metabolism.	(95)
REGULATION OF AEROBIC RESPIRATION	131	(1) Regulating the level of glucose metabolism. (2) Reactive oxygen species (ROS) are a byproduct of aerobic respiration and signaling molecules, which controls various cellular functions.	(96, 97)
CELLULAR RESPONSE TO OXYGEN CONTAINING COMPOUND	104	Disorder of glucose and lipid metabolism is an important cause for the development of T2DM.	(102)
AEROBIC RESPIRATION	101	Regulating energy metabolism,and then affecting T2DM.	(98)
REGULATION OF GLYCOLYTIC PROCESS	98	Producing energy and inducing mitochondrial dysfunction and oxidative stress.	(99)
REGULATION OF DNA BINDING	91	Regulating the function of mitochondrial.	(100)

The top 10 KEGG pathways are shown in **Tables 5**, **S3**. The most enriched KEGG pathway was “Parkinson’s Disease” (enriched in 1213 shortest paths). It has been reported that Parkinson’s disease (PD) and T2DM have common pathological mechanisms (76–78, 111). For example, oxidative stress and mitochondrial dysfunction are involved in both T2DM and PD pathogenesis (77). Strikingly, there are also a series of common biological pathways in T2DM, PD and cancer, such as mitochondrial dysfunction and protein homeostasis (112). Furthermore, the most significant KEGG pathway with the minimum FDR was “Leishmania Infection” (FDR=0.0000629) (**Figure 3B**), indicating the inflammatory response in the immune system (82, 113, 114). Notably, the inflammatory response is also often closely related to cancer (113). The classical aging pathway, the “mTOR signaling pathway” was also enriched in shortest pathway (**Figure S2**), indicating the interrelationship between oxidative aging and T2DM.

The top 10 BP terms are shown in **Tables 6**, **S4**. For example, the top enriched BP term was “Regulation of aerobic respiration” (enriched in 29 shortest paths), which was related to energy and mitochondrial function (96). In addition, reactive oxygen species (ROS) are byproducts of aerobic respiration that control various cellular functions (97). The BP term with the minimum FDR was “Regulation of DNA binding” (FDR=0.0000282) (**Figure 3A**), which is vital to T2DM by dysregulating mitochondria and energy metabolism (100). Obviously, the accumulation of DNA damage is also a hallmark of cancer (115). Overall, these results identified various aspects of risk factors for T2DM, such as oxidative stress, aging, energy metabolism and immune systems.

### 2.5 Network markers revealed key mechanisms between aging and T2DM

Network markers were identified by calculating the betweenness in the shortest path of each ‘‘oxidative-disease’’ pair, where the top markers are shown in **Table 7**. For example, the top network marker was SCD (stearyl-coenzyme A desaturase), which is mainly expressed in adipose tissue and can catalyze the synthesis of monounsaturated fatty acids (116). In addition, SCD can affect lipid metabolism and mediate steroidogenesis, playing an important role in insulin resistance (117, 118). Furthermore, SCD participates in mediating the inflammatory reaction, which promotes the progression of cancer (119). Moreover, there were also a series of shortest paths through SITR1 (**Figure S3**, where permutation p-value=0.002 and 0, before and after sensitive analysis), which was as a clssical aging marker. Thus, network markers indicate the crucial role of oxidative stress dysfunction, along with energy metabolism, in T2DM.

**Table 7:** The top 10 genes with the highest number before and after sensitive analysis.

Before sensitive analysis			After sensitive analysis		
Gene Symbol	Betweenness	P-value	Gene Symbol	Betweenness	P-value
SCD	3403	0	SCD	355	0
MARCKSL1	2049	0	MRPL11	325	0
APOD	1910	0	ATOX1	300	0
FOS	1827	0	COX7A2	223	0
ATOX1	1462	0	FOS	212	0
PCGF2	1288	0	NENF	164	0
COX7A2	1266	0	ISCU	163	0
MRPL11	1135	0	COX4I1	151	0
OGT	1098	0	HYAL2	146	0
NDUFA8	10003	0	MMP9	101	0

### 2.6 Pan-cancer analysis further verified the mechanism of oxidative aging in T2DM

**Figure 4:**
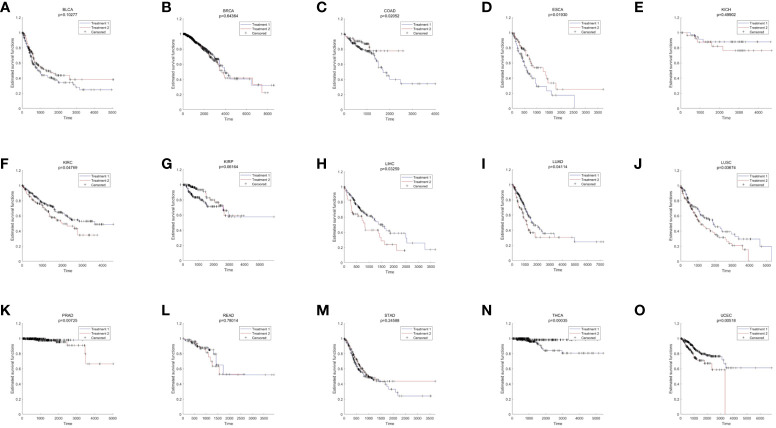
The results of survival analysis across different cancer types. **(A)** BLCA; **(B)** BRCA; **(C)** COAD; **(D)** ESCA; **(E)** KICH; **(F)** KIRC; **(G)** KIRP; **(H)** LIHC; **(I)** LUAD; **(J)** LUSC; **(K)** PRAD; **(L)** READ; **(M)** STAD; **(N)** THCA; **(O)** LIHC.

Pan-cancer analysis was used to further verify the relative functions of T2DM oxidative aging markers in cancer. For example, oxidative aging markers in the integrated model were used to evaluate the survival index across different cancer types. There were 9 out of 15 cancer types with significant results (including COAD, ESCA, KIRC, LIHC, LUAD, LUSC, PRAD, THCA and UCEC, shown in **Figure 4**). These results suggest that oxidative aging markers can also be used as relative risk factors in cancer.

**Figure 5:**
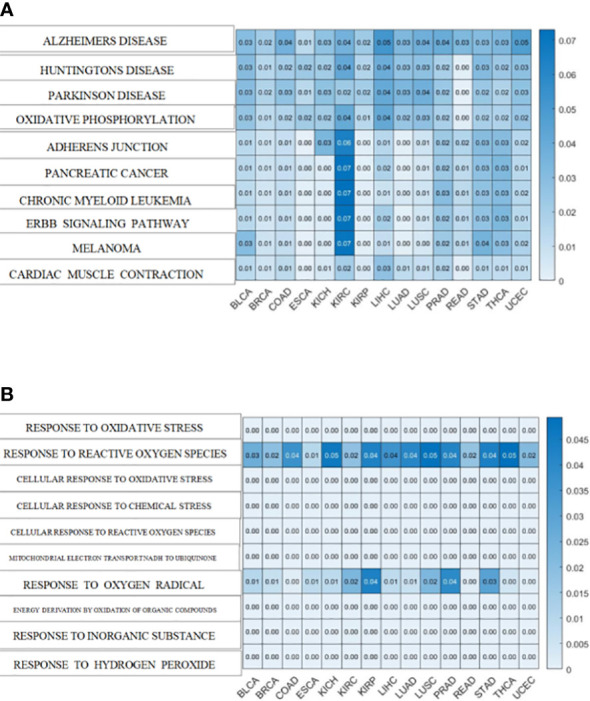
The enrichment analysis shared by cancers. **(A)**KEGG pathways enriched in 15 cancers **(B)**. BP terms enriched in 15 cancers.

**Figure 6:**
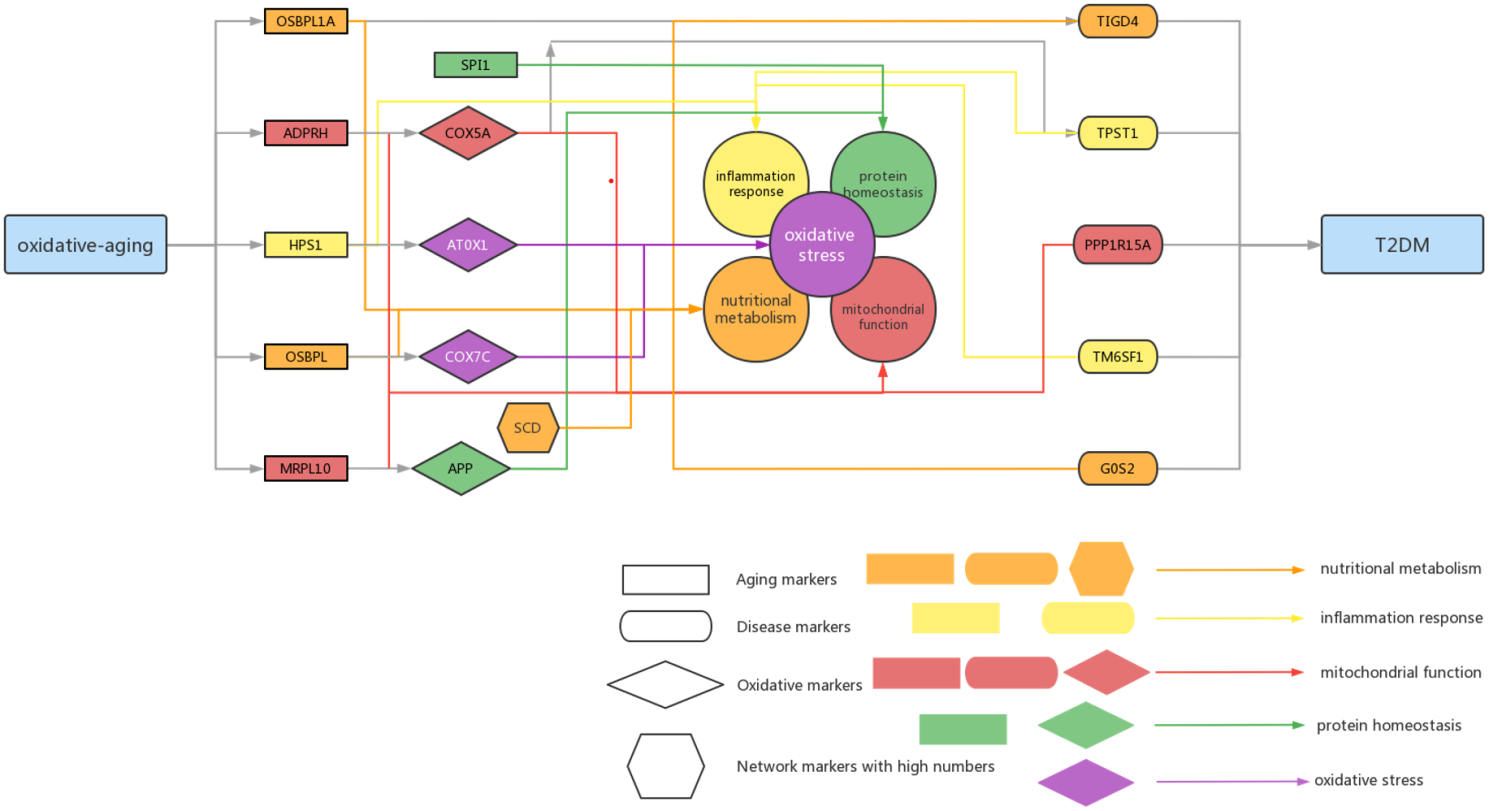
Summarized mechanisms of oxidative-aging in T2DM Rectangle genes represent aging markers, oval genes represent disease markers, rhombus genes represent oxidative markers, hexagon genes represent network markers with high numbers. Orange arrows indicate the gene involved in nutritional metabolism, yellow arrows indicate the gene involved in inflammation response, red arrows indicate the gene associated with mitochondrial function, green arrows indicate the gene associated with protein homeostasis.

Additionally, both the commonality and specificity across 15 cancer types were investigated based on enrichment analysis. The top 10 common KEGG pathways are shown in **Figures 5**, **S4**, where “Alzheimer’s Disease” was the top KEGG pathway. Alzheimer’s disease (AD) and cancer share common risk factors. For example, aging is one of the greatest risk factors for the development of Alzheimer’s disease, and the risk of cancer also increases with increasing age (120). In addition, some cancer patients may have a higher risk of Alzheimer’s disease (121). **Figures 6**, **S2** showed the top 10 common BP terms in 15 cancers. “Regulation of cellular respiration” was the top BP term, indicating the key role of energy metabolism in cancer (122). Cellular respiration participates in energy metabolism and is also a hallmark of many cancers (123). The specific enrichment results within each cancer are also summarized in **Tables 8**, **9**, **S6**, **S7** (112, 120–163), indicating a series of oxidative aging-related risk factors in cancer, such as the inflammatory response, energy metabolism and mitochondrial function. Overall, our results highlighted a series of crucial functions related to oxidative aging, which can also be used to study potential mechanisms in cancer.

**Table 8:** KEGG pathways in each cancer with the minimum FDR.

Type of cancer	FDR	KEGG	Functions	Reference
BLCA	8.72e-05	ERBB SIGNALING PATHWAY	Related to human cancer pathogenesis.	(124)
BLCA	8.72e-05	PROGESTERONE MEDIATED OOCYTE MATURATION	The source of immune cells and macrophages.	(125)
BLCA	8.72e-05	PANCREATIC CANCER	A fatal malignancy with an aggressive disease course.	(126)
BRCA	7.82e-06	INTESTINAL IMMUNE NETWORK FOR IGA PRODUCTION	Related to immune system.	(127)
COAD	9.79e-06	HUNTINGTONS DISEASE	Cancer and Huntington’s Disease have common pathogenesis.	(128)
ESCA	1.08e-07	PARKINSONS DISEASE	Parkinson Disease and cancer share some common biological pathways, such as mitochondrial dysfunction and protein homeostasis.	(112)
KICH	7.64e-07	OXIDATIVE PHOSPHORYLATION	Cancer cells utilize certain pathways to enhance oxidative phosphorylation.	(129)
KICH	7.64e-07	PARKINSONS DISEASE	Parkinson Disease and cancer share some common biological pathways, such as mitochondrial dysfunction and protein homeostasis.	(112)
KIRC	1.47e-05	RENAL CELL CARCINOMA	Main factor contributed to kidney cancer.	(130)
KIRC	1.47e-05	MELANOMA	The most lethal form of skin cancer.	(131)
KIRP	4.32e-07	ALZHEIMERS DISEASE	(1) age is the risk factor for the development of Alzheimer’s Disease and cancer. (2) some cancer patients may have a higher risk of Alzheimer’s Disease.	(120, 121)
LIHC	1.31e-04	ERBB SIGNALING PATHWAY	Related to human cancer pathogenesis.	(124)
LIHC	1.31e-04	PANCREATIC CANCER	A fatal malignancy with an aggressive disease course.	(126)
LUAD	5.99e-08	OXIDATIVE PHOSPHORYLATION	Cancer cells utilize certain pathways to enhance oxidative phosphorylation.	(129)
LUAD	5.99e-08	PARKINSONS DISEASE	Parkinson Disease and cancer share some common biological pathways, such as mitochondrial dysfunction and protein homeostasis.	(112)
LUSC	2.54e-05	BLADDER CANCER	The ninth most common malignancy worldwide.	(132)
PRAD	2.13e-04	OXIDATIVE PHOSPHORYLATION	Cancer cells utilize certain pathways to enhance oxidative phosphorylation.	(129)
PRAD	2.13e-04	PARKINSONS DISEASE	Parkinson Disease and cancer share some common biological pathways, such as mitochondrial dysfunction and protein homeostasis.	(112)
READ	4.88e-05	ADHERENS JUNCTION	Downregulation of E-cadherin, the two major components of adherens junctions, and p120, is a frequently recurrent hallmark of carcinomas.	(133)
READ	4.88e-05	GLIOMA	The most malignant and aggressive form of brain tumors, accounting for the majority of brain cancer-related deaths.	(134)
READ	4.88e-05	MELANOMA	The most lethal form of skin cancer.	(131)
STAD	2.94e-05	MELANOMA	The most lethal form of skin cancer.	(131)
THCA	5.08e-05	GAP JUNCTION	Genetic or acquired alterations of connexin proteins have been implicated in cancer.	(135)
UCEC	6.41e-06	ALZHEIMERS DISEASE	(1) age is the risk factor for the development of Alzheimer’s Disease and cancer. (2) some cancer patients may have a higher risk of Alzheimer’s Disease.	(120, 121)

**Table 9:** BP terms in each cancer with the minimum FDR.

Type of cancer	FDR	BP	Functions	Reference
BLCA	7.53e-07	NEGATIVE REGULATION OF INSULIN SECRETION INVOLVED IN CELLULAR RESPONSE TO GLUCOSE STIMULUS	Creating conditions that force cancer cells to rely more on metabolites and limited factors.	(136)
BRCA	2.09e-05	REGULATION OF OXIDATIVE PHOSPHORYLATION	Playing a crucial role in cancer progression.	(137)
BRCA	2.09e-05	RESPONSE TO HEPATOCYTE GROWTH FACTOR	The Cancer cell growth, survival, and migration of cancer cell are relied on an HGF-dependent manner.	(138)
COAD	9.24e-07	CELLULAR RESPONSE TO CADMIUM ION	Cadmium is an established carcinogen in both humans and animals.	(139)
ESCA	9.12e-07	NEGATIVE REGULATION OF PROTEIN CATABOLIC PROCESS	Playing dual roles in tumorigenesis and cancer progression.	(140)
KICH	2.36e-06	RESPONSE TO HYDROGEN PEROXIDE	The progression of cancer is related to effect of hydrogen peroxide.	(141)
KIRC	2.86e-05	RESPONSE TO IMMOBILIZATION STRESS	Enhancing the ability of some cancer cells to enter a dormant state.	(142)
KIRP	1.76e-06	MITOCHONDRIAL ELECTRON TRANSPORT NADH TO UBIQUINONE	Cancer cell propagation is closely related to the regulation of the electron transport chain.	(143)
LIHC	3.87e-07	CELLULAR RESPONSE TO HYDROGEN PEROXIDE	Regulating catalase expression to target the redox state of cancer cells.	(144)
LUAD	2.37e-07	ELECTRON TRANSPORT CHAIN	Electrons originating from different metabolic processes are guided into the mitochondrial electron transport chain (ETC) to drive the oxidative phosphorylation process.	(145)
LUSC	2.74e-05	CELLULAR RESPIRATION	Tumors gain energy mainly from glucose to lactate and only partially through cellular respiration involving oxygen.	(146)
PRAD	3.42e-04	RESPONSE TO OXIDATIVE STRESS	Related to cancer, which can regulate the progression of cancer.	(147)
READ	2.02e-05	CELLULAR RESPONSE TO REACTIVE OXYGEN SPECIES	ROS dynamically affect the tumor microenvironment, and are known to initiate cancer angiogenesis, metastasis, and survival at various concentrations.	(148)
STAD	6.00e-05	POSITIVE REGULATION OF CYTOSOLIC CALCIUM ION CONCENTRATION	Cancer cell proliferation and apoptosis depend on the intracellular Ca (2 +) concentration.	(149)
THCA	7.46e-05	REGULATION OF NUCLEOCYTOPLASMIC TRANSPORT	The nucleocytoplasmic transport of macromolecules is critical for both cellular physiology and pathology, playing an important role in the treatment of cancer.	(150)
UCEC	2.10e-07	AEROBIC RESPIRATION	Alterations in cancer glucose metabolism include leading to a shift in metabolism from aerobic respiration to glycolysis.	(151)

## 3 Discussion

It is well known that aging-related oxidative stress plays a crucial role in T2DM (3). However, the essential relationship among aging, oxidative stress and T2DM still needs to be explored in more depth. In this paper, a series of computational methods were performed to explore these relationships in T2DM as well as the relative mechanisms. First, both the aging model and disease model were optimized, and relative aging markers and disease markers were identified. Next, the integrated oxidative aging model was built to identify essential “aging-oxidative-disease” relationships. Finally, network analysis, enrichment analysis, sensitivity analysis and pan-cancer analysis were used to further explore the potential mechanisms between oxidative aging and T2DM. As a result, various risk factors in T2DM were integrated.

Our results highlighted that energy metabolism was vital to the development of T2DM. For example, the integrated oxidative aging model identified a series of key markers in T2DM that were closely related to energy metabolism. OSBPL1A and T1GD4 participate in nutritional metabolism; the former is mainly involved in lipid metabolism and cholesterol metabolism, and the latter is mainly related to glycogen metabolism (32–34). ADPRH and PPP1R15A can lead to energy metabolism imbalance (35, 63). COX5A can affect mitochondrial function, and ATOX1 is the redox catalyst, both of which can affect energy metabolism through mitochondrial dysfunction (39, 65). Furthermore, as the top network marker, SCD is mainly expressed in adipose tissue and can catalyze the synthesis of monounsaturated fatty acids (116). It can affect lipid metabolism and mediate steroidogenesis, which plays an important role in insulin resistance (117, 118). SIRT1 was also identified by calculating the betweenness. In MCMC, the greatest difference in the absolute value pair was “OSBPL7-COX7C-TM6SF1”, where OSBPL7 participates in lipid binding and transport (49, 50) and COX7C is related to cellular respiration as a potential biomarker of diabetes (51, 52). The classical energy metabolism pathway, “mTOR signaling pathway”, was also identified using the enrichment analysis, indicating the key interaction between oxidative aging and T2DM.

Protein homeostasis is also involved in the progression of T2DM. For instance, amyloid precursor protein (APP) is an oxidative marker identified by MCMC that promotes the secretion of amyloid proteins (164). SPI1 (Spi-1 Proto-Oncogene) was involved in the negative regulation of protein, which caused restraint of aerobic glycolysis (165) (**Figure 3**). In summary, both APP and SPI1 are related to protein homeostasis and even accelerate the development of both T2DM and neurodegenerative diseases (NDs). That is, protein homeostasis is a common mechanism in both T2DM and ND (166, 167).

The inflammatory response also plays an important role in the development of T2DM. For example, the aging marker HPS1 affects the biogenesis of lysosome-associated cellular organelles and even participates in regulating cellular inflammation (61, 62). The disease marker TPST1 induces the secretion of some cytokines, along with the inflammatory response (37, 38). TM6SF1, as one of the key markers identified by MCMC, was involved in transmembrane transport in macrophages, thus highlighting the key role of the immune system in T2DM (53).

Furthermore, there are a series of experiments and relative clinical stastic results also revealed significant relationships between the identified oxidative aging markers and T2DM. For example, it has been reported that *in vitro* oxidative stress in mammalian skeletal muscle leads to substantial insulin resistance to distal insulin signaling and glucose transport activity (p=9.2e-05) (168). Chronic oxidative stress can also leads to decreased responsiveness to insulin, ultimately leading to diabetes reported by Alina Berdichevsky et al (p=0.01) (169). Besides, NFKBIA affects the wound healing in diabetic foot ulceration (DFU) (p=0.006) (170), MYC and SCD are related to pyroptosis and immune infiltration in T2DM (p=0.001) (171). The experiment of Parker C. Wilson et al using single-nucleus RNA sequencing has been revealed that GCH1 is associated with early-stage diabetic nephropathy (p=4.88e-09) (172) In short, our results also presented key clinical indices with the help of the integrated oxidative model.

T2DM is associated with an increased risk of developing cancers, such as COAD, PRAD, and THCA (30). It is well known that T2DM and cancer have common risk factors, such as oxidative stress, energy metabolism, inflammation and protein homeostasis (22, 23, 173). Our results also proved that inflammation and energy metabolism were common risk factors in cancers, and even survival analysis further verified the key role of oxidative aging markers across different cancer types. Oxidative stress may lead to chronic inflammation, which in turn can induce most chronic diseases, including both cancer and T2DM. In addition, oxidative stress can damage the normal function of mitochondria as well as energy metabolism, which plays an important role in the development of T2DM and cancer. In short, various risk factors related to oxidative aging were also confirmed in cancer.

According to the oxi-inflamm-aging theory, the aging process is regulated by chronic oxidative stress, as well as the inflammatory response (174). It is well known that dysregulated oxidative stress triggers a series of signaling pathways, thus leading to pancreatic beta cell damage (175). In addition, the cellular senescence theory also highlights cellular inflammation and the oxidative stress response during the aging process (176, 177). That is, cellular senescence may also play an important role in the pathogenesis of T2DM (i.e., through the mTOR signaling pathway) (177, 178). Furthermore, these risk factors even interact with each other and then promote T2DM. For example, the imbalance of energy metabolism could interact with a series of pathways, such as lipid accumulation, chronic inflammation and insulin resistance, triggering T2DM progression (179). It has been reported that normal homeostasis in the insulin-driven immunometabolic network is vital to the preservation of insulin sensitivity in healthy aging (180). Here, our work also highlighted the interaction between the immune system and energy metabolism in the development of T2DM (**Figure 3**; **Tables 5**, **6**), which is also crucial in cancer (**Figures 4**, **5**). With the help of the integrated oxidative aging model, our study revealed that oxidative stress was interrelated with various aging-related risk factors in T2DM (**Tables 2**–**6**), such as the inflammatory response, mitochondrial function and protein homeostasis. These results further confirmed both the oxi-inflamm-aging and cellular senescence theories. Overall, potential aging-related mechanisms in T2DM were integrated in the context of oxidative stress (**Figure 6**).

## 4 Conclusion

In this study, machine learning was performed to predict aging and T2DM, and then relative biomarkers were identified. An integrated oxidative aging model was built to explore the essential relationship between oxidative aging and T2DM. The key roles of nutritional metabolism, the inflammatory response, mitochondrial function and protein homeostasis in T2DM were highlighted in our work with the help of sensitivity analysis, enrichment analysis, network analysis and pan-cancer analysis. In conclusion, various risk factors were integrated in the development of T2DM as well as cancer based on oxidative aging.

## 5 Materials and methods

### 5.1 Data and preprocessing

All gene expression data were downloaded from the Gene Expression Omnibus (GEO) database (https://www.ncbi.nlm.nih.gov/geo/), including GSE362, GSE15790, GSE18732, GSE29221, GSE29226, GSE29231, GSE37171, GSE38642, GSE76894, and GSE182120. These datasets were from eight different platforms: GPL96, GPL97, GPL8450, GPL9486, GPL6947, GPL570, GPL6244, and GPL17586.

The gene expression profiles were processed as follows:

(1) Only the samples with both the age and phenotype index (i.e., type 2 diabetes versus control) were retained; otherwise, they were deleted.
(2) The gene expression matrix for each dataset was integrated by summarizing the probe number within the gene symbol.
(3) The total data matrix was integrated, and the missing gene expression values were filled with values of 0.
(4) Genes with missing values ≥ 30% were deleted.
(5) The gene expression matrix was transformed by logarithmic transformation if it contained outliers.
(6) Based on the mean and the standard deviation of gene expression for control individuals, the z-score normalization was performed for both T2DM and control samples.
(7) The singular value decomposition (SVD) method was performed to eliminate the intersample variation based on the top three principal components of the control samples.
(8) The z score was then utilized to normalize all samples based on the mean and the standard deviation of the control samples.
(9) The training set and the test set were randomly divided according to a ratio of approximately 2:1.

As a result, a total of 489 samples were obtained, including 208 samples of healthy aged people (age > 50 years old, 145 training datasets + 63 test datasets), 131 samples of healthy young people (age ≤ 50, 90 + 41), 110 samples of T2DM aged people (age > 50, 75 + 35) and 40 samples of T2DM young people (age ≤ 50, 25 + 15), containing 12958 gene symbols (**Tables S1**–**S3**).

We also obtained paired gene expression (RNAseq) profiles (“Batch effects normalized mRNA data”) and clinical data from the TCGA database through the xena platform (https://xenabrowser.net/hub/). Cancer types with ≥10 adjacent normal samples were retained. As a result, there were 15 cancer types used in this work: BLCA (408 cancer samples and 19 adjacent normal samples), BRCA(1102 + 113), COAD(451 + 41), ESCA(185 + 11), KICH(66 + 25), KIRC(534 + 72), KIRP(291 + 32), LIHC(376 + 50), LUAD(517 + 59), LUSC(504 + 51), PRAD(498 + 52), READ(160 + 10), STAD(414 + 35), THCA(513 + 59) and UCEC(533 + 22). The tumor expression profiles from the same patient were averaged. Genes with missing values ≥30% were deleted.

### 5.2 Modeling the aging model and disease model

After randomization as well as a random disorder, the healthy population samples were divided into a training dataset and a test dataset. The ratio of training dataset samples to test dataset samples was close to 2:1. The ReliefF algorithm was used to select key features, and then the first 500 models were studied to train predictors. The optimal model was selected by 10-fold cross-validation. To verify the accuracy of the aging predictor, the selected model was verified in the test dataset.

(1) In the aging model, the normal aged group (age>50) was labeled 1, and the young healthy group (age ≤ 50) was labeled 0; in the disease model, the T2DM group was labeled 1, and the control group (age ≤ 50) was labeled 0.
(2) The 12958 genes were sorted by the ReliefF algorithm;
(3) The predictor was generated using the k-nearest neighbor (kNN, k=3, correlation distance) algorithm. The optimal model was selected by 10-fold cross-validation, where the model with the highest accuracy rate was chosen.
(4) The identified features were considered aging and disease markers. As a result, 304 aging markers and 299 disease markers were identified.

### 5.2 Identifying essential relationships in T2DM by an integrated oxidative aging model

The integrated oxidative aging model was built to identify the essential relationship among aging, oxidative stress and T2DM. The computational pipeline was referred to by Mendelian randomization (MR), although it was not as strict as MR (**Figure 1C**).

In this model, the aging-related oxidative stress markers were considered oxidative aging markers, where the relative aging/disease markers were identified in “Methods 5.2”. As a result, the essential relationships among aging, oxidative stress and disease (T2DM) markers were identified as key “aging-oxidative-disease” triples in T2DM.

MR is a statistical method for assessing the causal relationship between risk factors and outcomes based on observational data (181, 182). The causal relationships between the instrumental variables, risk factors, and outcome variables were assessed as follows.

(1) There was a correlation between the instrumental variable and the risk factor.
(2) There was no correlation between the instrumental variable and the confounding factor.
(3) There was no correlation between the instrumental variable and the outcome variable after deleting the effect from the risk factor.

Here, the aging marker was used as the auxiliary variable (similar to the instrumental variable in MR), and the oxidative stress markers were used as the candidate risk factor. Then, aging-related oxidative (“oxidative aging”) markers were identified as the risk factor, and disease markers were used as the outcome variable. That is, the integrated oxidative aging model aimed to explore essential relationships among aging, oxidative stress and disease markers in T2DM. This model was performed as follows:

(1) Oxidative markers were obtained as candidate risk factors based on Biological Processes (BP) of Gene Ontology (GO) through the Gene Set Enrichment Analysis (GSEA) platform (http://www.gsea-msigdb.org/gsea/downloads.jsp, “OXIDATIVE” was taken as the keyword). As a result, 310 candidate oxidative markers were selected.

(2) The correlation (differential coexpression) pattern was used to select aging markers that strongly correlated with candidate oxidative stress markers with the help of the Kruskal−Wallis test. Here, the differential coexpression was calculated as follows:


(1) p=Kruskal−Wallis test (aging_marker.∗oxidative_marker, phenotype)

where the phenotype could be defined as 1 (T2DM) and 0 (control).

Furthermore, both a p-value<0.05 and Benjamini−Hochberg false discovery rate (FDR)<0.1 were used to select strongly correlated aging markers.

(3) To reduce the correlation between the auxiliary variable (aging marker) and confounding factors, as well as further select a strong correlation between the aging marker and the candidate oxidative marker, a permutation test was performed by generating the simulated aging markers from the same number of randomly selected markers to each candidate oxidative marker; this process was repeated 1000 times, and then the p-value was calculated as the proportion of occurrence times (larger than the real mean difference) of the absolute difference between T2DM and control in 1000 permutations. The relationship between each aging marker and the candidate oxidative marker was retained if the permutation P<0.05.

(4) Correlation (differential coexpression) was used to select oxidative markers that strongly correlated with disease markers with the help of the Kruskal−Wallis test. Here, the differential coexpression was calculated as follows:


(2) p=Kruskal−Wallis test (oxidative_marker.∗disease_marker, phenotype)

where the phenotype could be defined as 1 (T2DM) and 0 (control).

Furthermore, both a p-value<0.05 and Benjamini−Hochberg false discovery rate (FDR)<0.1 were used to select strongly correlated oxidative markers.

(5) To reduce the correlation between the risk factor (oxidative marker) and confounding factors, as well as further select a strong correlation between the oxidative marker and the disease marker, a permutation test was performed by generating the simulated oxidative markers from the same number of randomly selected markers to each disease marker; this process was repeated 1000 times, and then the p-value was calculated as the proportion of occurrence times (larger than the real mean difference) of the absolute difference between T2DM and control in 1000 permutations. The relationship between each aging marker and the candidate oxidative marker was retained if the permutation P<0.05.

(6) The direct relationships for any other factors (genes) were found to reduce the correlation between the auxiliary variable (aging marker) and confounding factors. If there was another factor (gene) that was directly correlated (differentially coexpressed) to both the aging marker and the disease marker, then the relationship from aging to disease was deleted.


(3) p=Kruskal−Wallis test (aging_marker.∗other_gene, phenotype)

(4) p=Kruskal−Wallis test (disease_marker.∗other_gene, phenotype)

where the phenotype could be defined as 1 (T2DM) and 0 (control).

Furthermore, both a p-value<0.05 and Benjamini−Hochberg false discovery rate (FDR)<0.1 were used to filter out any direct relationships.

(7) To filter out the effect of horizontal pleiotropy, the aging–disease relationship was further examined by comparing the correlation between each aging and disease marker, through the oxidative marker or otherwise. Herein, steps ①–③ were used to calculate the correlations between auxiliary variables and outcome variables without the background of the risk factor, and step ④ was used to calculate the correlations between auxiliary variables and outcome variables with the context of the risk factor.

① The residual of each disease marker (“residual A”) was calculated based on the oxidative marker:


(5) $$\text{residual}\_A=disease\_mar\ker-b_{1}*oxidative\_mar\ker$$

where *b_1_* is the regression coefficient.

② The residual of each aging marker (“residual B”) was calculated based on the oxidative marker:


(6) $$\text{residual}\_B=aging\_mar\ker-b_{2}*oxidative\_mar\ker$$

where *b_2_* is the regression coefficient.

③ The abovementioned two residuals were further compared, and the residual of the disease marker was calculated (as “residual C”):


(7) $$\text{residual}\_C=residual\_A-b_{3}*residual-\_B$$

where *b_3_* is the regression coefficient.

④ The residual of the disease marker (“residual D”) was calculated based on the aging marker.

⑤ The difference (between “residual C” and “residual D”) was tested between the T2DM and control subgroups using the Kruskal–Wallis test (P<0.05 and FDR<0.1).

Finally, the essential relationship among the aging marker, oxidative marker and disease marker was retained. Thus, 11829 “aging-oxidative-disease” triples were identified, including 105 aging markers, 83 oxidative markers and 282 disease markers. Thus, these 83 oxidative markers were used as oxidative aging markers (risk factors), and 282 disease markers were also used to discriminate the T2DM phenotype.

### 5.4 Sensitivity analysis using the MCMC method

To further explore the relationship among aging, oxidative stress and T2DM, sensitivity analysis was performed based on the Markov chain Monte Carlo (MCMC) method, where “aging-oxidative-disease” triples identified by MR were further evaluated as a candidate relationship. The MCMC method is used to sample certain posterior distributions in a high-dimensional space based on a given probabilistic background. The key step of MCMC is to construct a Markov chain whose equilibrium distribution is equal to the target probability distribution. The steps were as follows:

(1) Constructing the transfer cores of the ergodic Markov chain. The prior distribution of each parameter was normally distributed based on all identified markers in each group (i.e., T2DM and control), respectively.

(2) Simulate the chains until equilibrium is reached. The Metropolis−Hastings sampling method was used to determine whether the new sample (θ *) was acceptable based on the α value.


(8) $$\alpha =\frac{\text{P}(\theta^{*}|X)*\text{q}(\theta^{n}\to \theta^{*})}{\text{P}(\theta^{n}|\text{X})*\text{q}(\theta^{n}\to \theta^{*})}$$

where *P* (*θ ^n^ | X*) and *P* (θ * | X) are the posterior probability of the nth accepted sample, the new sample *q* (θ ^n^ → θ *) is the transition probability from the nth accepted sample to the new sample, and *q* (θ * → θ ^n^) is the transition probability from the new sample to the *n-th* accepted sample.

In this work, the disease score was used to evaluate the simulated samples, with 1000 random samples used as candidate samples for each group (i.e., T2DM or control). The disease score was calculated by comparing the distance between normal and T2DM training samples based on the 282 disease markers identified by the integrated oxidative aging model:


(9) $$\text{disease}\_\text{score}=\sum_{\text{k}=1}^{7}\text{distance}\_\text{of}\_\text{neareast}\_\text{neighbour}\_\text{in}\_\text{control}-\sum_{\text{k}=1}^{7}\text{distance}\_\text{of}\_\text{neareast}\_\text{neighbour}\_\text{in}\_\text{T}2\text{DM}$$

(3) Performing the global sensitivity analysis

The correlation index was used to evaluate each “aging-oxidative-disease” triple in the accepted samples (including both T2DM and control):


(10) $$\text{correlation}\_\text{index}=\frac{\text{disease}\_\text{marker}-\text{aging}\_\text{marker}}{\text{oxidative}\_\text{marker}-\text{aging}\_\text{marker}}$$

As a result, the correlation index was calculated in each “aging-oxidative-disease” triple for all accepted samples. Then, the Kruskal–Wallis test was used to evaluate each correlation index in each “aging-oxidative-disease” triple, where p-value<0.05 and FDR<0.1 were set as the threshold. Finally, 2501 “aging-oxidative-disease” triples were identified as sensitive relationships, including 41 aging markers, 37 oxidative markers and 61 disease markers.

### 5.5 Constructing the differential coexpression network

To further reveal the relationship between “oxidative aging” and T2DM, a differential coexpression network was constructed by the following steps:

(1) The Pearson correlation coefficient for each pair of genes was calculated based on the T2DM and control groups.
(2) The Benjamini−Hochberg FDR method was used to adjust the p-values of the correlation coefficient.
(3) The relationship between each gene pair was retained if the coefficient value in T2DM had the opposite sign (i.e., + or -) to that in control, as well as p< 0.05 and FDR< 0.1.
(4) The shortest path between each pair of oxidative aging and disease markers was selected based on the differential coexpression network using the Dijkstra algorithm.

### 5.6 Enrichment analysis

The gene functions were further explored by enrichment analysis of the shortest pathway. Gene Ontology (GO) terms and KEGG pathways for the GSEA platform were obtained from gene set enrichment analysis (http://software.broadinstitute.org/gsea/downloads.jsp, version 7.5). The hypergeometric distribution was used to test the degree of enrichment of the GO BP and KEGG pathways. Hypergeometric test formula:


(11) $$P\left(X\geq x\right)=1-\sum_{k=0}^{x-1}\frac{C_{M}^{k}\times C_{N-M}^{n-k}}{C_{M}^{k}}$$

where *N* is the total number of genes in the gene set, *M* is the number of known genes (such as KEGG pathway or BP terms), which is the number of genes identified in each shortest pathway, and *k* is the number of common genes between known genes and candidate genes identified in each “oxidative-disease” shortest pathway. The p-value of each path was controlled using the Benjamin-Hochberg method. Finally, pathways with p<0.05 and FDR<0.1 were retained.

### 5.7 Identifying network markers

The subnetwork with the shortest pathways among the selected “oxidative-disease” pairs was constructed, and genes in the subnetwork were sorted by their betweennesses in descending order. To test whether the top betweenness genes were hubs in the background network, we ran a permutation to count the occurrence time of the top genes in the shortest paths between randomly selected genes (containing the same numbers of “oxidative-disease” pairs, based on the identified “aging-oxidative-disease” triples) when they had greater betweennesses than those in our study. We repeated this process 1000 times, and the p-value was calculated as the proportion of occurrence times of the top betweenness genes in 1000 permutations.

### 5.8 Pan-cancer analysis

The survival analysis was performed based on the oxidative aging markers (identified by the integrated oxidative aging model in 5.3) for each cancer using the Kaplan−Meier method. The tumor samples of each cancer were divided into two groups based on the mean value of the oxidative aging markers. Then, the Kaplan−Meier method was used to evaluate the survival difference between these two groups, and the significance was estimated by the log-rank test. A p-value<0.05 was considered statistically significant.

Genes were considered differentially expressed if they satisfied the following criteria:

(1) Fold change>2;
(2) p-value<0.05 in the Kruskal−Wallis test;
(3) Benjamin-Hochberg false discovery rate (FDR)<0.1.

Then, the differential expression networks were constructed for each cancer, where the details were also the same as 5.5. As a result, each shoreat pathway was selected from each pair of oxidative aging markers and differentially expressed genes (as disease markers in cancer) using the Dijkstra algorithm. Furthermore, enrichment analysis was performed by the “oxidative-disease” shortest pathway for each cancer type, where both p<0.05 and FDR<0.1 were used.

## Data availability statement

The original contributions presented in the study are included in the article/**Supplementary Material**. Further inquiries can be directed to the corresponding authors.

## Author contributions

LX, LL, and YW designed the study. YC, LY, SZ and YW analyzed the data. YC, LY and YW interpreted the results. YC, MX, SR and YW visualized the results. All authors wrote and revised the manuscript. All authors contributed to the article and approved the submitted version.

## Supplementary material

Supplementary Table 1 The detailed datasets used in this work.

Supplementary Table 2 The gene symbols used in this work.

## References

[1] Sun H Saeedi P Karuranga S Pinkepank M Ogurtsova K Duncan BB. IDF diabetes atlas: global, regional and country-level diabetes prevalence estimates for 2021 and projections for 2045. Diabetes Res Clin Pract (2022) 183:109119. doi: 10.1016/j.diabres.2021.109119 34879977 PMC11057359

[2] Artasensi A Pedretti A Vistoli G Fumagalli L. Type 2 diabetes mellitus: a review of multi-target drugs. Molecules (2020) 25(8):1987. doi: 10.3390/molecules25081987 32340373 PMC7221535

[3] Gunasekaran U Gannon M. Type 2 diabetes and the aging pancreatic beta cell. Aging (Albany NY). (2011) 3(6):565–75. doi: 10.18632/aging.100350 PMC3164365 21765202

[4] Pengpid S Peltzer K. Prevalence and correlates of undiagnosed, diagnosed, and total type 2 diabetes among adults in Morocco, 2017. Sci Rep (2022) 12(1):16092. doi: 10.1038/s41598-022-20368-4 36168026 PMC9515107

[5] Jura M Kozak LP. Obesity and related consequences to ageing. Age (Dordr). (2016) 38(1):23. doi: 10.1007/s11357-016-9884-3 26846415 PMC5005878

[6] van den Beld AW Kaufman JM Zillikens MC Lamberts SWJ Egan JM van der Lely AJ. The physiology of endocrine systems with ageing. Lancet Diabetes Endocrinol (2018) 6(8):647–58. doi: 10.1016/S2213-8587(18)30026-3 PMC6089223 30017799

[7] Diniz Pereira J Gomes Fraga V Morais Santos AL Carvalho MDG Caramelli P Braga Gomes K. Alzheimer's disease and type 2 diabetes mellitus: a systematic review of proteomic studies. J Neurochem (2021) 156(6):753–76. doi: 10.1111/jnc.15166 32909269

[8] Dal Canto E Ceriello A Rydén L Ferrini M Hansen TB Schnell O. Diabetes as a cardiovascular risk factor: an overview of global trends of macro and micro vascular complications. Eur J Prev Cardiol (2019) 26(2_suppl):25–32. doi: 10.1177/2047487319878371 31722562

[9] van Eersel ME Joosten H Gansevoort RT Dullaart RP Slaets JP Izaks GJ. The interaction of age and type 2 diabetes on executive function and memory in persons aged 35 years or older. PloS One (2013) 8(12):e82991. doi: 10.1371/journal.pone.0082991 24367577 PMC3867457

[10] Gallagher EJ LeRoith D. Obesity and diabetes: the increased risk of cancer and cancer-related mortality. Physiol Rev (2015) 95(3):727–48. doi: 10.1152/physrev.00030.2014 PMC4491542 26084689

[11] Frijhoff J Winyard PG Zarkovic N Davies SS Stocker R Cheng D. Clinical relevance of biomarkers of oxidative stress. Antioxid Redox Signal (2015) 23(14):1144–70. doi: 10.1089/ars.2015.6317 PMC4657513 26415143

[12] Sies H. Oxidative stress: a concept in redox biology and medicine. Redox Biol (2015) 4:180–3. doi: 10.1016/j.redox.2015.01.002 PMC4309861 25588755

[13] Giacco F Brownlee M. Oxidative stress and diabetic complications. Circ Res (2010) 107(9):1058–70. doi: 10.1161/CIRCRESAHA.110.223545 PMC2996922 21030723

[14] Beckman KB Ames BN. The free radical theory of aging matures. Physiol Rev (1998) 78(2):547–81. doi: 10.1152/physrev.1998.78.2.547 9562038

[15] Golden TR Hinerfeld DA Melov S. Oxidative stress and aging: beyond correlation. Aging Cell (2002) 1(2):117–23. doi: 10.1046/j.1474-9728.2002.00015.x 12882341

[16] Oliveira BF Nogueira-Machado JA Chaves MM. The role of oxidative stress in the aging process. Sci World J (2010) 10:1121–8. doi: 10.1100/tsw.2010.94 PMC5763815 20563535

[17] Mancini A Di Segni C Raimondo S Olivieri G Silvestrini A Meucci E. Thyroid hormones, oxidative stress, and inflammation. Mediators Inflamm (2016) 2016:6757154. doi: 10.1155/2016/6757154 27051079 PMC4802023

[18] Hussain T Tan B Yin Y Blachier F Tossou MC Rahu N. Oxidative stress and inflammation: what polyphenols can do for us? Oxid Med Cell Longev (2016) 2016:7432797. doi: 10.1155/2016/7432797 27738491 PMC5055983

[19] Grevendonk L Connell NJ McCrum C Fealy CE Bilet L Bruls YMH. Impact of aging and exercise on skeletal muscle mitochondrial capacity, energy metabolism, and physical function. Nat Commun (2021) 12(1):4773. doi: 10.1038/s41467-021-24956-2 34362885 PMC8346468

[20] Lennicke C Cochemé HM. Redox metabolism: ROS as specific molecular regulators of cell signaling and function. Mol Cell (2021) 81(18):3691–707. doi: 10.1016/j.molcel.2021.08.018 34547234

[21] Tramunt B Smati S Grandgeorge N Lenfant F Arnal JF Montagner A. Sex differences in metabolic regulation and diabetes susceptibility. Diabetologia (2020) 63(3):453–61. doi: 10.1007/s00125-019-05040-3 PMC6997275 31754750

[22] Reuter S Gupta SC Chaturvedi MM Aggarwal BB. Oxidative stress, inflammation, and cancer: how are they linked? Free Radic Biol Med (2010) 49(11):1603–16. doi: 10.1016/j.freeradbiomed.2010.09.006 PMC2990475 20840865

[23] Tan YT Lin JF Li T Li JJ Xu RH Ju HQ. LncRNA-mediated posttranslational modifications and reprogramming of energy metabolism in cancer. Cancer Commun (Lond). (2021) 41(2):109–20. doi: 10.1002/cac2.12108 PMC7896749 33119215

[24] Zhou X Du HH Jiang M Zhou C Deng Y Long X. Antioxidant effect of lactobacillus fermentum CQPC04-fermented soy milk on d-Galactose-Induced oxidative aging mice. Front Nutr (2021) 8:727467. doi: 10.3389/fnut.2021.727467 34513906 PMC8429822

[25] Alaa AM Bolton T Di Angelantonio E Rudd JHF van der Schaar M. Cardiovascular disease risk prediction using automated machine learning: a prospective study of 423,604 UK biobank participants. PloS One (2019) 14(5):e0213653. doi: 10.1371/journal.pone.0213653 31091238 PMC6519796

[26] Tran KA Kondrashova O Bradley A Williams ED Pearson JV Waddell N. Deep learning in cancer diagnosis, prognosis and treatment selection. Genome Med (2021) 13(1):152. doi: 10.1186/s13073-021-00968-x 34579788 PMC8477474

[27] Deberneh HM Kim I. Prediction of type 2 diabetes based on machine learning algorithm. Int J Environ Res Public Health (2021) 18(6):3317. doi: 10.3390/ijerph18063317 33806973 PMC8004981

[28] Dagliati A Marini S Sacchi L Cogni G Teliti M Tibollo V. Machine learning methods to predict diabetes complications. J Diabetes Sci Technol (2018) 12(2):295–302. doi: 10.1177/1932296817706375 28494618 PMC5851210

[29] Li Z Pan X Cai YD. Identification of type 2 diabetes biomarkers from mixed single-cell sequencing data with feature selection methods. Front Bioeng Biotechnol (2022) 10:890901. doi: 10.3389/fbioe.2022.890901 35721855 PMC9201257

[30] Pearson-Stuttard J Papadimitriou N Markozannes G Cividini S Kakourou A Gill D. Type 2 diabetes and cancer: an umbrella review of observational and mendelian randomization studies. Cancer Epidemiol Biomarkers Prev (2021) 30(6):1218–28. doi: 10.1158/1055-9965.EPI-20-1245 PMC9398112 33737302

[31] Swerdlow DI. Mendelian randomization and type 2 diabetes. Cardiovasc Drugs Ther (2016) 30(1):51–7. doi: 10.1007/s10557-016-6638-5 PMC4789194 26809778

[32] Wang Z Wang F. Identification of ten-gene related to lipid metabolism for predicting overall survival of breast invasive carcinoma. Contrast Media Mol Imaging (2022) 2022:8348780. doi: 10.1155/2022/8348780 35919504 PMC9293542

[33] Tao JH Wang XT Yuan W Chen JN Wang ZJ Ma YB. Reduced serum high-density lipoprotein cholesterol levels and aberrantly expressed cholesterol metabolism genes in colorectal cancer. World J Clin Cases (2022) 10(14):4446–59. doi: 10.12998/wjcc.v10.i14.4446 PMC9125299 35663062

[34] Fiuza-Luces C Santos-Lozano A Llavero F Campo R Nogales-Gadea G Díez-Bermejo J. Muscle molecular adaptations to endurance exercise training are conditioned by glycogen availability: a proteomics-based analysis in the McArdle mouse model. J Physiol (2018) 596(6):1035–61. doi: 10.1113/JP275292 PMC5851888 29315579

[35] Gong YY Liu YY Li J Su L Yu S Zhu XN. Hypermethylation of Cox5a promoter is associated with mitochondrial dysfunction in skeletal muscle of high fat diet-induced insulin resistant rats. PloS One (2014) 9(12):e113784. doi: 10.1371/journal.pone.0113784 25436770 PMC4249960

[36] Peng L Ma W Xie Q Chen B. Identification and validation of hub genes for diabetic retinopathy. PeerJ (2021) 9:e12126. doi: 10.7717/peerj.12126 34603851 PMC8445088

[37] Ting TW Brett MS Tan ES Shen Y Lee SP Lim EC. Cockayne syndrome due to a maternally-inherited whole gene deletion of ERCC8 and a paternally-inherited ERCC8 exon 4 deletion. Gene (2015) 572(2):274–8. doi: 10.1016/j.gene.2015.07.065 26210811

[38] Akasheh RT Pini M Pang J Fantuzzi G. Increased adiposity in annexin A1-deficient mice. PloS One (2013) 8(12):e82608. doi: 10.1371/journal.pone.0082608 24312665 PMC3846785

[39] Nechushtan A Smith CL Lamensdorf I Yoon SH Youle RJ. Bax and bak coalesce into novel mitochondria-associated clusters during apoptosis. J Cell Biol (2001) 153(6):1265–76. doi: 10.1083/jcb.153.6.1265 PMC2192024 11402069

[40] Nagao M Esguerra JLS Asai A Ofori JK Edlund A Wendt A. Potential protection against type 2 diabetes in obesity through lower CD36 expression and improved exocytosis in β-cells. Diabetes (2020) 69(6):1193–205. doi: 10.2337/db19-0944 PMC7243297 32198214

[41] Zhao Z Zhang X Zhao C Choi J Shi J Song K. Protection of pancreatic beta-cells by group VIA phospholipase A(2)-mediated repair of mitochondrial membrane peroxidation. Endocrinology (2010) 151(7):3038–48. doi: 10.1210/en.2010-0016 PMC2903934 20463052

[42] Heemskerk MM Giera M Bouazzaoui FE Lips MA Pijl H van Dijk KW. Increased PUFA content and 5-lipoxygenase pathway expression are associated with subcutaneous adipose tissue inflammation in obese women with type 2 diabetes. Nutrients (2015) 7(9):7676–90. doi: 10.3390/nu7095362 PMC4586557 26378572

[43] Ivashchenko O Van Veldhoven PP Brees C Ho YS Terlecky SR Fransen M. Intraperoxisomal redox balance in mammalian cells: oxidative stress and interorganellar cross-talk. Mol Biol Cell (2011) 22(9):1440–51. doi: 10.1091/mbc.E10-11-0919 PMC3084667 21372177

[44] Rack JGM Ariza A Drown BS Henfrey C Bartlett E Shirai T. (ADP-ribosyl)hydrolases: structural basis for differential substrate recognition and inhibition. Cell Chem Biol (2018) 25(12):1533–1546.e12. doi: 10.1016/j.chembiol.2018.11.001 30472116 PMC6309922

[45] Zhang C Wang L Liu H Deng G Xu P Tan Y. ADPRH is a prognosis-related biomarker and correlates with immune infiltrates in low grade glioma. J Cancer (2021) 12(10):2912–20. doi: 10.7150/jca.51643 PMC8040889 33854592

[46] Ouyang Yb Lane WS Moore KL. Tyrosylprotein sulfotransferase: purification and molecular cloning of an enzyme that catalyzes tyrosine O-sulfation, a common posttranslational modification of eukaryotic proteins. Proc Natl Acad Sci USA (1998) 95(6):2896–901. doi: 10.1073/pnas.95.6.2896 PMC19666 9501187

[47] Westmuckett AD Moore KL. Lack of tyrosylprotein sulfotransferase activity in hematopoietic cells drastically attenuates atherosclerosis in ldlr-/- mice. Arterioscler Thromb Vasc Biol (2009) 29(11):1730–6. doi: 10.1161/ATVBAHA.109.192963 PMC2923564 19679829

[48] Xiyang YB Liu R Wang XY Li S Zhao Y Lu BT. COX5A plays a vital role in memory impairment associated with brain aging via the BDNF/ERK1/2 signaling pathway. Front Aging Neurosci (2020) 12:215. doi: 10.3389/fnagi.2020.00215 32754029 PMC7365906

[49] Aharon-Hananel G Romero-Afrima L Saada A Mantzur C Raz I Weksler-Zangen S. Cytochrome c oxidase activity as a metabolic regulator in pancreatic beta-cells. Cells (2022) 11(6):929. doi: 10.3390/cells11060929 35326380 PMC8946064

[50] Karslioglu E Kleinberger JW Salim FG Cox AE Takane KK Scott DK. cMyc is a principal upstream driver of beta-cell proliferation in rat insulinoma cell lines and is an effective mediator of human beta-cell replication. Mol Endocrinol (2011) 25(10):1760–72. doi: 10.1210/me.2011-1074 PMC3182418 21885567

[51] Huard K Londregan AT Tesz G Bahnck KB Magee TV Hepworth D. Discovery of selective small molecule inhibitors of monoacylglycerol acyltransferase 3. J Med Chem (2015) 58(18):7164–72. doi: 10.1021/acs.jmedchem.5b01008 26258602

[52] Rouault TA Tong WH. Iron-sulfur cluster biogenesis and human disease. Trends Genet (2008) 24(8):398–407. doi: 10.1016/j.tig.2008.05.008 18606475 PMC2574672

[53] Heitzer T Krohn K Albers S Meinertz T. Tetrahydrobiopterin improves endothelium-dependent vasodilation by increasing nitric oxide activity in patients with type II diabetes mellitus. Diabetologia (2000) 43(11):1435–8. doi: 10.1007/s001250051551 11126415

[54] Gu C Liu S Wang H Dou H. Role of the thioredoxin interacting protein in diabetic nephropathy and the mechanism of regulating NOD-like receptor protein 3 inflammatory corpuscle. Int J Mol Med (2019) 43(6):2440–50. doi: 10.3892/ijmm.2019.4163 PMC6488169 31017263

[55] Pinto-Junior DC Silva KS Michalani ML Yonamine CY Esteves JV Fabre NT. Advanced glycation end products-induced insulin resistance involves repression of skeletal muscle GLUT4 expression. Sci Rep (2018) 8(1):8109. doi: 10.1038/s41598-018-26482-6 29802324 PMC5970140

[56] Chou CW Hsieh YH Ku SC Shen WJ Anuraga G Khoa Ta HD. Potential prognostic biomarkers of OSBPL family genes in patients with pancreatic ductal adenocarcinoma. Biomedicines (2021) 9(11):1601. doi: 10.3390/biomedicines9111601 34829830 PMC8615799

[57] Wright MB Varona Santos J Kemmer C Maugeais C Carralot JP Roever S. Compounds targeting OSBPL7 increase ABCA1-dependent cholesterol efflux preserving kidney function in two models of kidney disease. Nat Commun (2021) 12(1):4662. doi: 10.1038/s41467-021-24890-3 34341345 PMC8329197

[58] Krishna S Arrojo E Drigo R Capitanio JS Ramachandra R Ellisman M Hetzer MW. Identification of long-lived proteins in the mitochondria reveals increased stability of the electron transport chain. Dev Cell (2021) 56(21):2952–65. doi: 10.1016/j.devcel.2021.10.008 PMC8664086 34715012

[59] Wang X Wang LT Yu B. UBE2D1 and COX7C as potential biomarkers of diabetes-related sepsis. BioMed Res Int (2022) 2022:9463717. doi: 10.1155/2022/9463717 35445133 PMC9015863

[60] Zeng Z Yu J Yang Z Du K Chen Y Zhou L. Investigation of M2 macrophage-related gene affecting patients prognosis and drug sensitivity in non-small cell lung cancer: evidence from bioinformatic and experiments. Front Oncol (2022) 12:1096449. doi: 10.3389/fonc.2022.1096449 36591493 PMC9797692

[61] Ahn EH Kim DW Shin MJ Ryu EJ Yong JI Chung SY. Tat-ATOX1 inhibits streptozotocin-induced cell death in pancreatic RINm5F cells and attenuates diabetes in a mouse model. Int J Mol Med (2016) 38(1):217–24. doi: 10.3892/ijmm.2016.2599 27222268

[62] Jiang A Gao H Kelley MR Qiao X. Inhibition of APE1/Ref-1 redox activity with APX3330 blocks retinal angiogenesis *in vitro* and *in vivo* . Vision Res (2011) 51(1):93–100. doi: 10.1016/j.visres.2010.10.008 20937296 PMC3010438

[63] Moreno-Gonzalez I Edwards Iii G Salvadores N Shahnawaz M Diaz-Espinoza R Soto C. Molecular interaction between type 2 diabetes and alzheimer's disease through cross-seeding of protein misfolding. Mol Psychiatry (2017) 22(9):1327–34. doi: 10.1038/mp.2016.230 PMC5495631 28044060

[64] Wu Z Chen A Zhang G Liu C Yin S Song R. ALDH3B1 protects interfollicular epidermal cells against lipid peroxidation via the NRF2 pathway. Cell Stress Chaperones (2022) 27(6):703–15. doi: 10.1007/s12192-022-01306-9 PMC9672232 36327089

[65] Pei-Yuan Z Yu-Wei L Xiang-Nan Z Song T Rong Z Xiao-Xiao H. Overexpression of axl reverses endothelial cells dysfunction in high glucose and hypoxia. J Cell Biochem (2019) 120(7):11831–41. doi: 10.1002/jcb.28462 30848518

[66] Yu N Fang X Zhao D Mu Q Zuo J Ma Y. Anti-diabetic effects of jiang tang xiao ke granule via PI3K/Akt signalling pathway in type 2 diabetes KKAy mice. PloS One (2017) 12(1):e0168980. doi: 10.1371/journal.pone.0168980 28045971 PMC5207690

[67] Pan X Mota S Zhang B. Circadian clock regulation on lipid metabolism and metabolic diseases. Adv Exp Med Biol (2020) 1276:53–66. doi: 10.1007/978-981-15-6082-8_5 32705594 PMC8593891

[68] Tian D Xiang Y Tang Y Ge Z Li Q Zhang Y. Circ-ADAM9 targeting PTEN and ATG7 promotes autophagy and apoptosis of diabetic endothelial progenitor cells by sponging mir-20a-5p. Cell Death Dis (2020) 11(7):526. doi: 10.1038/s41419-020-02745-x 32661238 PMC7359341

[69] Anderson KA Lin F Ribar TJ Stevens RD Muehlbauer MJ Newgard CB. Deletion of CaMKK2 from the liver lowers blood glucose and improves whole-body glucose tolerance in the mouse. Mol Endocrinol (2012) 26(2):281–91. doi: 10.1210/me.2011-1299 PMC3275166 22240810

[70] Carmona-Rivera C Simeonov DR Cardillo ND Gahl WA Cadilla CL. A divalent interaction between HPS1 and HPS4 is required for the formation of the biogenesis of lysosome-related organelle complex-3 (BLOC-3). Biochim Biophys Acta (2013) 1833(3):468–78. doi: 10.1016/j.bbamcr.2012.10.019 PMC3556189 23103514

[71] Tang H Huang X Pang S. Regulation of the lysosome by sphingolipids: potential role in aging. J Biol Chem (2022) 298(7):102118. doi: 10.1016/j.jbc.2022.102118 35691340 PMC9257404

[72] Patel V Bidault G Chambers JE Carobbio S Everden AJT Garcés C. Inactivation of Ppp1r15a minimises weight gain and insulin resistance during caloric excess in female mice. Sci Rep (2019) 9(1):2903. doi: 10.1038/s41598-019-39562-y 30814564 PMC6393541

[73] Zhao Y Li W Zhang K Xu M Zou Y Qiu X. Revealing oxidative stress-related genes in osteoporosis and advanced structural biological study for novel natural material discovery regarding MAPKAPK2. Front Endocrinol (Lausanne) (2022) 13:1052721. doi: 10.3389/fendo.2022.1052721 36479222 PMC9720258

[74] Hatori Y Lutsenko S. The role of copper chaperone Atox1 in coupling redox homeostasis to intracellular copper distribution. Antioxidants (Basel) (2016) 5(3):25. doi: 10.3390/antiox5030025 27472369 PMC5039574

[75] Hatori Y Lutsenko S. An expanding range of functions for the copper chaperone/antioxidant protein Atox1. Antioxid Redox Signal (2013) 19(9):945–57. doi: 10.1089/ars.2012.5086 PMC3763234 23249252

[76] Hassan A Sharma Kandel R Mishra R Gautam J Alaref A Jahan N. Diabetes mellitus and parkinson's disease: shared pathophysiological links and possible therapeutic implications. Cureus (2020) 12(8):e9853. doi: 10.7759/cureus.9853 32832307 PMC7437092

[77] Cheong JLY de Pablo-Fernandez E Foltynie T Noyce AJ. The association between type 2 diabetes mellitus and parkinson's disease. J Parkinsons Dis (2020) 10(3):775–89. doi: 10.3233/JPD-191900 PMC7458510 32333549

[78] Chohan H Senkevich K Patel RK Bestwick JP Jacobs BM Bandres Ciga S. Type 2 diabetes as a determinant of parkinson's disease risk and progression. Mov Disord (2021) 36(6):1420–9. doi: 10.1002/mds.28551 PMC9017318 33682937

[79] Pugazhenthi S Qin L Reddy PH. Common neurodegenerative pathways in obesity, diabetes, and alzheimer's disease. Biochim Biophys Acta Mol Basis Dis (2017) 1863(5):1037–45. doi: 10.1016/j.bbadis.2016.04.017 PMC5344771 27156888

[80] Burillo J Marqués P Jiménez B González-Blanco C Benito M Guillén C. Insulin resistance and diabetes mellitus in alzheimer's disease. Cells (2021) 10(5):1236. doi: 10.3390/cells10051236 34069890 PMC8157600

[81] Kreider RB Kalman DS Antonio J Ziegenfuss TN Wildman R Collins R. International society of sports nutrition position stand: safety and efficacy of creatine supplementation in exercise, sport, and medicine. J Int Soc Sports Nutr (2017) 14:18. doi: 10.1186/s12970-017-0173-z 28615996 PMC5469049

[82] Akhoundi M Downing T Votýpka J Kuhls K Lukeš J Cannet A. Leishmania infections: molecular targets and diagnosis. Mol Aspects Med (2017) 57:1–29. doi: 10.1016/j.mam.2016.11.012 28159546

[83] Ashayeri Ahmadabad R Mirzaasgari Z Gorji A Khaleghi Ghadiri M. Toll-like receptor signaling pathways: novel therapeutic targets for cerebrovascular disorders. Int J Mol Sci (2021) 22(11):6153. doi: 10.3390/ijms22116153 34200356 PMC8201279

[84] Yu GH Li SF Wei R Jiang Z. Diabetes and colorectal cancer risk: clinical and therapeutic implications. J Diabetes Res (2022) 2022:1747326. doi: 10.1155/2022/1747326 35296101 PMC8920658

[85] Dissanayake WC Sorrenson B Shepherd PR. The role of adherens junction proteins in the regulation of insulin secretion. Biosci Rep (2018) 38(2):BSR20170989. doi: 10.1042/BSR20170989 29459424 PMC5861323

[86] Zikherman J Au-Yeung B. The role of T cell receptor signaling thresholds in guiding T cell fate decisions. Curr Opin Immunol (2015) 33:43–8. doi: 10.1016/j.coi.2015.01.012 PMC4397149 25660212

[87] Nolfi-Donegan D Braganza A Shiva S. Mitochondrial electron transport chain: Oxidative phosphorylation, oxidant production, and methods of measurement. Redox Biol (2020) 37:101674. doi: 10.1016/j.redox.2020.101674 32811789 PMC7767752

[88] Kolwicz SC Jr Purohit S Tian R. Cardiac metabolism and its interactions with contraction, growth, and survival of cardiomyocytes. Circ Res (2013) 113(5):603–16. doi: 10.1161/CIRCRESAHA.113.302095 PMC3845521 23948585

[89] Zheng X Narayanan S Xu C Eliasson Angelstig S Grünler J Zhao A. Repression of hypoxia-inducible factor-1 contributes to increased mitochondrial reactive oxygen species production in diabetes. Elife (2022) 11:e70714. doi: 10.7554/eLife.70714 35164902 PMC8846593

[90] Leenders F Groen N de Graaf N Engelse MA Rabelink TJ de Koning EJP. Oxidative stress leads to β-cell dysfunction through loss of β-cell identity. Front Immunol (2021) 12:690379. doi: 10.3389/fimmu.2021.690379 34804002 PMC8601632

[91] Walton EL. Oxidative stress and diabetes: glucose response in the cROSsfire. BioMed J (2017) 40(5):241–4. doi: 10.1016/j.bj.2017.10.001 PMC6138607 29179878

[92] He L He T Farrar S Ji L Liu T Ma X. Antioxidants maintain cellular redox homeostasis by elimination of reactive oxygen species. Cell Physiol Biochem (2017) 44(2):532–53. doi: 10.1159/000485089 29145191

[93] Ray PD Huang BW Tsuji Y. Reactive oxygen species (ROS) homeostasis and redox regulation in cellular signaling. Cell Signal (2012) 24(5):981–90. doi: 10.1016/j.cellsig.2012.01.008 PMC3454471 22286106

[94] Simaan H Lev S Horwitz BA. Oxidant-sensing pathways in the responses of fungal pathogens to chemical stress signals. Front Microbiol (2019) 10:567. doi: 10.3389/fmicb.2019.00567 30941117 PMC6433817

[95] Levonen AL Hill BG Kansanen E Zhang J Darley-Usmar VM. Redox regulation of antioxidants, autophagy, and the response to stress: implications for electrophile therapeutics. Free Radic Biol Med (2014) 71:196–207. doi: 10.1016/j.freeradbiomed.2014.03.025 24681256 PMC4042208

[96] Orang AV Petersen J McKinnon RA Michael MZ. Micromanaging aerobic respiration and glycolysis in cancer cells. Mol Metab (2019) 23:98–126. doi: 10.1016/j.molmet.2019.01.014 30837197 PMC6479761

[97] Kasai S Shimizu S Tatara Y Mimura J Itoh K. Regulation of Nrf2 by mitochondrial reactive oxygen species in physiology and pathology. Biomolecules (2020) 10(2):320. doi: 10.3390/biom10020320 32079324 PMC7072240

[98] Toyoda Y Saitoh S. Adaptive regulation of glucose transport, glycolysis and respiration for cell proliferation. Biomol Concepts (2015) 6(5-6):423–30. doi: 10.1515/bmc-2015-0018 26418646

[99] Rabbani N Thornalley PJ. Hexokinase-2 glycolytic overload in diabetes and ischemia-reperfusion injury. Trends Endocrinol Metab (2019) 30(7):419–31. doi: 10.1016/j.tem.2019.04.011 31221272

[100] Klinge CM. Estrogenic control of mitochondrial function. Redox Biol (2020) 31:101435. doi: 10.1016/j.redox.2020.101435 32001259 PMC7212490

[101] Zhang J Wang X Vikash V Ye Q Wu D Liu Y. ROS and ROS-mediated cellular signaling. Oxid Med Cell Longev (2016) 2016:4350965. doi: 10.1155/2016/4350965 26998193 PMC4779832

[102] Xu L Li Y Yin L Qi Y Sun H Sun P. miR-125a-5p ameliorates hepatic glycolipid metabolism disorder in type 2 diabetes mellitus through targeting of STAT3. Theranostics (2018) 8(20):5593–609. doi: 10.7150/thno.27425 30555566 PMC6276304

[103] Coulthard LR White DE Jones DL McDermott MF Burchill SA. p38(MAPK): stress responses from molecular mechanisms to therapeutics. Trends Mol Med (2009) 15(8):369–79. doi: 10.1016/j.molmed.2009.06.005 PMC3016890 19665431

[104] Popa A Georgescu M Popa SG Nica AE Georgescu EF. New insights in the molecular pathways linking obesity, type 2 diabetes and cancer. Rom J Morphol Embryol (2019) 60(4):1115–25. 32239086

[105] Hüttemann M Lee I Samavati L Yu H Doan JW. Regulation of mitochondrial oxidative phosphorylation through cell signaling. Biochim Biophys Acta (2007) 1773(12):1701–20. doi: 10.1016/j.bbamcr.2007.10.001 18240421

[106] Andrae J Gallini R Betsholtz C. Role of platelet-derived growth factors in physiology and medicine. Genes Dev (2008) 22(10):1276–312. doi: 10.1101/gad.1653708 PMC2732412 18483217

[107] Drouin M Saenz J Chiffoleau E. C-type lectin-like receptors: head or tail in cell death immunity. Front Immunol (2020) 11:251. doi: 10.3389/fimmu.2020.00251 32133013 PMC7040094

[108] Ivanova EA Orekhov AN. Monocyte activation in immunopathology: cellular test for development of diagnostics and therapy. J Immunol Res (2016) 2016:4789279. doi: 10.1155/2016/4789279 26885534 PMC4739459

[109] Coulthard MG Morgan M Woodruff TM Arumugam TV Taylor SM Carpenter TC. Eph/Ephrin signaling in injury and inflammation. Am J Pathol (2012) 181(5):1493–503. doi: 10.1016/j.ajpath.2012.06.043 23021982

[110] Boekema EJ Braun HP. Supramolecular structure of the mitochondrial oxidative phosphorylation system. J Biol Chem (2007) 282(1):1–4. doi: 10.1074/jbc.R600031200 17102127

[111] Picca A Guerra F Calvani R Romano R Coelho-Júnior HJ Bucci C. Mitochondrial dysfunction, protein misfolding and neuroinflammation in parkinson's disease: roads to biomarker discovery. Biomolecules (2021) 11(10):1508. doi: 10.3390/biom11101508 34680141 PMC8534011

[112] Ejma M Madetko N Brzecka A Guranski K Alster P Misiuk-Hojło M. The links between parkinson's disease and cancer. Biomedicines (2020) 8(10):416. doi: 10.3390/biomedicines8100416 33066407 PMC7602272

[113] Singh N Baby D Rajguru JP Patil PB Thakkannavar SS Pujari VB. Inflammation and cancer. Ann Afr Med (2019) 18(3):121–6. doi: 10.4103/aam.aam_56_18 PMC6704802 31417011

[114] Daryabor G Atashzar MR Kabelitz D Meri S Kalantar K. The effects of type 2 diabetes mellitus on organ metabolism and the immune system. Front Immunol (2020) 11:1582. doi: 10.3389/fimmu.2020.01582 32793223 PMC7387426

[115] Huang R Zhou PK. DNA Damage repair: historical perspectives, mechanistic pathways and clinical translation for targeted cancer therapy. Signal Transduct Target Ther (2021) 6(1):254. doi: 10.1038/s41392-021-00648-7 34238917 PMC8266832

[116] Ntambi JM Miyazaki M Stoehr JP Lan H Kendziorski CM Yandell BS. Loss of stearoyl-CoA desaturase-1 function protects mice against adiposity. Proc Natl Acad Sci USA (2002) 99(17):11482–6. doi: 10.1073/pnas.132384699 PMC123282 12177411

[117] Yuan X Hu S Li L Liu H He H Wang J. Metabolomic analysis of SCD during goose follicular development: implications for lipid metabolism. Genes (Basel) (2020) 11(9):1001. doi: 10.3390/genes11091001 32858946 PMC7565484

[118] Yuan X Abdul-Rahman I Hu S Li L He H Xia L. Mechanism of SCD participation in lipid droplet-mediated steroidogenesis in goose granulosa cells. Genes (Basel) (2022) 13(9):1516. doi: 10.3390/genes13091516 36140684 PMC9498882

[119] Tang B Qiu J Hu S Li L Wang J. Role of stearyl-coenzyme a desaturase 1 in mediating the effects of palmitic acid on endoplasmic reticulum stress, inflammation, and apoptosis in goose primary hepatocytes. Anim Biosci (2021) 34(7):1210–20. doi: 10.5713/ajas.20.0444 PMC8255868 33152216

[120] Valentine D Teerlink CC Farnham JM Rowe K Kaddas H Tschanz J. Comorbidity and cancer disease rates among those at high-risk for alzheimer's disease: a population database analysis. Int J Environ Res Public Health (2022) 19(24):16419. doi: 10.3390/ijerph192416419 36554301 PMC9778263

[121] Kesler SR Rao V Ray WJ Rao A. Alzheimer's disease neuroimaging initiative. probability of alzheimer's disease in breast cancer survivors based on gray-matter structural network efficiency. Alzheimers Dement (Amst) (2017) 9:67–75. doi: 10.1016/j.dadm.2017.10.002 29201992 PMC5700833

[122] Kroemer G Pouyssegur J. Tumor cell metabolism: cancer's achilles' heel. Cancer Cell (2008) 13(6):472–82. doi: 10.1016/j.ccr.2008.05.005 18538731

[123] Ojha R Tantray I Rimal S Mitra S Cheshier S Lu B. Regulation of reverse electron transfer at mitochondrial complex I by unconventional notch action in cancer stem cells. Dev Cell (2022) 57(2):260–276.e9. doi: 10.1016/j.devcel.2021.12.020 35077680 PMC8852348

[124] Arteaga CL Engelman JA. ERBB receptors: from oncogene discovery to basic science to mechanism-based cancer therapeutics. Cancer Cell (2014) 25(3):282–303. doi: 10.1016/j.ccr.2014.02.025 24651011 PMC4018830

[125] Dressing GE Goldberg JE Charles NJ Schwertfeger KL Lange CA. Membrane progesterone receptor expression in mammalian tissues: a review of regulation and physiological implications. Steroids (2011) 76(1-2):11–7. doi: 10.1016/j.steroids.2010.09.006 PMC3005015 20869977

[126] Goral V. Pancreatic cancer: pathogenesis and diagnosis. Asian Pac J Cancer Prev (2015) 16(14):5619–24. doi: 10.7314/apjcp.2015.16.14.5619 26320426

[127] Ko HJ Chang SY. Regulation of intestinal immune system by dendritic cells. Immune Netw (2015) 15(1):1–8. doi: 10.4110/in.2015.15.1.1 25713503 PMC4338263

[128] Zsindely N Siági F Bodai L. DNA Methylation in huntington's disease. Int J Mol Sci (2021) 22(23):12736. doi: 10.3390/ijms222312736 34884540 PMC8657460

[129] LeBleu VS O'Connell JT Gonzalez Herrera KN Wikman H Pantel K Haigis MC. PGC-1α mediates mitochondrial biogenesis and oxidative phosphorylation in cancer cells to promote metastasis. Nat Cell Biol (2014) 16(10):992–1003. doi: 10.1038/ncb3039 25241037 PMC4369153

[130] Gray RE Harris GT. Renal cell carcinoma: diagnosis and management. Am Fam Physician. (2019) 99(3):179–84. 30702258

[131] Davis LE Shalin SC Tackett AJ. Current state of melanoma diagnosis and treatment. Cancer Biol Ther (2019) 20(11):1366–79. doi: 10.1080/15384047.2019.1640032 PMC6804807 31366280

[132] Kader AK. Bladder cancer. Sci World J (2011) 11:2565–6. doi: 10.1100/2011/251920 PMC3253580 22235187

[133] Venhuizen JH Jacobs FJC Span PN Zegers MM. P120 and e-cadherin: double-edged swords in tumor metastasis. Semin Cancer Biol (2020) 60:107–20. doi: 10.1016/j.semcancer.2019.07.020 31369816

[134] Ludwig K Kornblum HI. Molecular markers in glioma. J Neurooncol (2017) 134(3):505–12. doi: 10.1007/s11060-017-2379-y PMC5568999 28233083

[135] Totland MZ Rasmussen NL Knudsen LM Leithe E. Regulation of gap junction intercellular communication by connexin ubiquitination: physiological and pathophysiological implications. Cell Mol Life Sci (2020) 77(4):573–91. doi: 10.1007/s00018-019-03285-0 PMC7040059 31501970

[136] Buono R Longo VD. Starvation, stress resistance, and cancer. Trends Endocrinol Metab (2018) 29(4):271–80. doi: 10.1016/j.tem.2018.01.008 PMC7477630 29463451

[137] Yu L Lu M Jia D Ma J Ben-Jacob E Levine H. Modeling the genetic regulation of cancer metabolism: interplay between glycolysis and oxidative phosphorylation. Cancer Res (2017) 77(7):1564–74. doi: 10.1158/0008-5472.CAN-16-2074 PMC5380541 28202516

[138] Owusu BY Galemmo R Janetka J Klampfer L. Hepatocyte growth factor, a key tumor-promoting factor in the tumor microenvironment. Cancers (Basel) (2017) 9(4):35. doi: 10.3390/cancers9040035 28420162 PMC5406710

[139] Hartwig A. Cadmium and cancer. Met Ions Life Sci (2013) 11:491–507. doi: 10.1007/978-94-007-5179-8_15 23430782

[140] Song Y Xu Y Pan C Yan L Wang ZW Zhu X. The emerging role of SPOP protein in tumorigenesis and cancer therapy. Mol Cancer (2020) 19(1):2. doi: 10.1186/s12943-019-1124-x 31901237 PMC6942384

[141] Vilema-Enríquez G Arroyo A Grijalva M Amador-Zafra RI Camacho J. Molecular and cellular effects of hydrogen peroxide on human lung cancer cells: potential therapeutic implications. Oxid Med Cell Longev (2016) 2016:1908164. doi: 10.1155/2016/1908164 27375834 PMC4916325

[142] Lam T Aguirre-Ghiso JA Geller MA Aksan A Azarin SM. Immobilization rapidly selects for chemoresistant ovarian cancer cells with enhanced ability to enter dormancy. Biotechnol Bioeng (2020) 117(10):3066–80. doi: 10.1002/bit.27479 PMC8505013 32589792

[143] Gao X Yang Y Wang J Zhang L Sun C Wang Y. Inhibition of mitochondria NADH-ubiquinone oxidoreductase (complex I) sensitizes the radioresistant glioma U87MG cells to radiation. BioMed Pharmacother (2020), 129:110460. doi: 10.1016/j.biopha.2020.110460 32768950

[144] Glorieux C Calderon PB. Catalase, a remarkable enzyme: targeting the oldest antioxidant enzyme to find a new cancer treatment approach. Biol Chem (2017) 398(10):1095–108. doi: 10.1515/hsz-2017-0131 28384098

[145] Raimondi V Ciccarese F Ciminale V. Oncogenic pathways and the electron transport chain: a dangeROS liaison. Br J Cancer (2020) 122(2):168–81. doi: 10.1038/s41416-019-0651-y PMC7052168 31819197

[146] Thorne JL Campbell MJ. Nuclear receptors and the warburg effect in cancer. Int J Cancer (2015) 137(7):1519–27. doi: 10.1002/ijc.29012 PMC4790452 24895240

[147] Ginckels P Holvoet P. Oxidative stress and inflammation in cardiovascular diseases and cancer: role of non-coding RNAs. Yale J Biol Med (2022) 95(1):129–52. PMC8961704 35370493

[148] Aggarwal V Tuli HS Varol A Thakral F Yerer MB Sak K. Role of reactive oxygen species in cancer progression: molecular mechanisms and recent advancements. Biomolecules (2019) 9(11):735. doi: 10.3390/biom9110735 31766246 PMC6920770

[149] Schwarz EC Qu B Hoth M. Calcium, cancer and killing: the role of calcium in killing cancer cells by cytotoxic T lymphocytes and natural killer cells. Biochim Biophys Acta (2013) 1833(7):1603–11. doi: 10.1016/j.bbamcr.2012.11.016 23220009

[150] Cautain B Hill R de Pedro N Link W. Components and regulation of nuclear transport processes. FEBS J (2015) 282(3):445–62. doi: 10.1111/febs.13163 PMC7163960 25429850

[151] Fogg VC Lanning NJ Mackeigan JP. Mitochondria in cancer: at the crossroads of life and death. Chin J Cancer (2011) 30(8):526–39. doi: 10.5732/cjc.011.10018 PMC3336361 21801601

[152] Law ML Metzger JM. Cardiac myocyte intrinsic contractility and calcium handling deficits underlie heart organ dysfunction in murine cancer cachexia. Sci Rep (2021) 11(1):23627. doi: 10.1038/s41598-021-02688-z 34880268 PMC8655071

[153] Amelio I Cutruzzolá F Antonov A Agostini M Melino G. Serine and glycine metabolism in cancer. Trends Biochem Sci (2014) 39(4):191–8. doi: 10.1016/j.tibs.2014.02.004 PMC3989988 24657017

[154] Liu J Wu Z Han D Wei C Liang Y Jiang T. Mesencephalic astrocyte-derived neurotrophic factor inhibits liver cancer through small ubiquitin-related modifier (SUMO)ylation-related suppression of NF-κB/Snail signaling pathway and epithelial-mesenchymal transition. Hepatology (2020) 71(4):1262–78. doi: 10.1002/hep.30917 PMC7187412 31469428

[155] Schuldt M Pei J Harakalova M Dorsch LM Schlossarek S Mokry M. Proteomic and functional studies reveal detyrosinated tubulin as treatment target in sarcomere mutation-induced hypertrophic cardiomyopathy. Circ Heart Fail (2021) 14(1):e007022. doi: 10.1161/CIRCHEARTFAILURE.120.007022 33430602 PMC7819533

[156] Faas M Ipseiz N Ackermann J Culemann S Grüneboom A Schröder F. IL-33-induced metabolic reprogramming controls the differentiation of alternatively activated macrophages and the resolution of inflammation. Immunity (2021) 54(11):2531–2546.e5. doi: 10.1016/j.immuni.2021.09.010 34644537 PMC7617137

[157] Ward MH Jones RR Brender JD de Kok TM Weyer PJ Nolan BT. Drinking water nitrate and human health: an updated review. Int J Environ Res Public Health (2018) 15(7):1557. doi: 10.3390/ijerph15071557 30041450 PMC6068531

[158] Vultaggio-Poma V Sarti AC Di Virgilio F. Extracellular ATP: a feasible target for cancer therapy. Cells (2020) 9(11):2496. doi: 10.3390/cells9112496 33212982 PMC7698494

[159] Martínez-Reyes I Cardona LR Kong H Vasan K McElroy GS Werner M. Mitochondrial ubiquinol oxidation is necessary for tumour growth. Nature (2020) 585(7824):288–92. doi: 10.1038/s41586-020-2475-6 PMC7486261 32641834

[160] Radin DP Tsirka SE. Interactions between tumor cells, neurons, and microglia in the glioma microenvironment. Int J Mol Sci (2020) 21(22):8476. doi: 10.3390/ijms21228476 33187183 PMC7698134

[161] Koike H Iwasawa K Ouchi R Maezawa M Giesbrecht K Saiki N. Modelling human hepato-biliary-pancreatic organogenesis from the foregut-midgut boundary. Nature (2019) 574(7776):112–6. doi: 10.1038/s41586-019-1598-0 PMC7643931 31554966

[162] Porporato PE Filigheddu N Pedro JMB Kroemer G Galluzzi L. Mitochondrial metabolism and cancer. Cell Res (2018) 28(3):265–80. doi: 10.1038/cr.2017.155 PMC5835768 29219147

[163] Zong WX Rabinowitz JD White E. Mitochondria and cancer. Mol Cell (2016) 61(5):667–76. doi: 10.1016/j.molcel.2016.02.011 PMC4779192 26942671

[164] Grimm MO Mett J Grimm HS Hartmann T Function APP. And lipids: a bidirectional link. Front Mol Neurosci (2017) 10:63. doi: 10.3389/fnmol.2017.00063 28344547 PMC5344993

[165] Wang J Wang X Guo Y Ye L Li D Hu A. Therapeutic targeting of SPIB/SPI1-facilitated interplay of cancer cells and neutrophils inhibits aerobic glycolysis and cancer progression. Clin Transl Med (2021) 11(11):e588. doi: 10.1002/ctm2.588 34841706 PMC8567044

[166] Sin O Nollen EA. Regulation of protein homeostasis in neurodegenerative diseases: the role of coding and non-coding genes. Cell Mol Life Sci (2015) 72(21):4027–47. doi: 10.1007/s00018-015-1985-0 PMC4605983 26190021

[167] Press M Jung T König J Grune T Höhn A. Protein aggregates and proteostasis in aging: amylin and β-cell function. Mech Ageing Dev (2019) 177:46–54. doi: 10.1016/j.mad.2018.03.010 29580826

[168] Archuleta TL Lemieux AM Saengsirisuwan V Teachey MK Lindborg KA Kim JS. Oxidant stress-induced loss of IRS-1 and IRS-2 proteins in rat skeletal muscle: role of p38 MAPK. Free Radic Biol Med (2009) 47(10):1486–93. doi: 10.1016/j.freeradbiomed.2009.08.014 PMC2767452 19703555

[169] Berdichevsky A Guarente L Bose A. Acute oxidative stress can reverse insulin resistance by inactivation of cytoplasmic JNK. J Biol Chem (2010) 285(28):21581–9. doi: 10.1074/jbc.M109.093633 PMC2898407 20430894

[170] Theocharidis G Thomas BE Sarkar D Mumme HL Pilcher WJR Dwivedi B. Single cell transcriptomic landscape of diabetic foot ulcers. Nat Commun (2022) 13(1):181. doi: 10.1038/s41467-021-27801-8 35013299 PMC8748704

[171] Song Y He C Jiang Y Yang M Xu Z Yuan L. Bulk and single-cell transcriptome analyses of islet tissue unravel gene signatures associated with pyroptosis and immune infiltration in type 2 diabetes. Front Endocrinol (Lausanne) (2023) 14:1132194. doi: 10.3389/fendo.2023.1132194 36967805 PMC10034023

[172] Wilson PC Wu H Kirita Y Uchimura K Ledru N Rennke HG. The single-cell transcriptomic landscape of early human diabetic nephropathy. Proc Natl Acad Sci USA (2019) 116(39):19619–25. doi: 10.1073/pnas.1908706116 PMC6765272 31506348

[173] Van Drie JH. Protein folding, protein homeostasis, and cancer. Chin J Cancer (2011) 30(2):124–37. doi: 10.5732/cjc.010.10162 PMC4013342 21272445

[174] Martínez de Toda I Ceprián N Díaz-Del Cerro E de la Fuente M. The role of immune cells in oxi-Inflamm-Aging. Cells (2021) 10(11):2974. doi: 10.3390/cells10112974 34831197 PMC8616159

[175] Luc K Schramm-Luc A Guzik TJ Mikolajczyk TP. Oxidative stress and inflammatory markers in prediabetes and diabetes. J Physiol Pharmacol (2019) 70(6):809–24. doi: 10.26402/jpp.2019.6.01 32084643

[176] Wu D Bi X Li P Xu D Qiu J Li K. Enhanced insulin-regulated phagocytic activities support extreme health span and longevity in multiple populations. Aging Cell (2023) 8:e13810. doi: 10.1111/acel.13810 PMC10186610 36883688

[177] Lananna BV Musiek ES. The wrinkling of time: aging, inflammation, oxidative stress, and the circadian clock in neurodegeneration. Neurobiol Dis (2020) 139:104832. doi: 10.1016/j.nbd.2020.104832 32179175 PMC7727873

[178] Saoudaoui S Bernard M Cardin GB Malaquin N Christopoulos A Rodier F. mTOR as a senescence manipulation target: a forked road. Adv Cancer Res (2021) 150:335–63. doi: 10.1016/bs.acr.2021.02.002 33858600

[179] Palmer AK Gustafson B Kirkland JL Smith U. Cellular senescence: at the nexus between ageing and diabetes. Diabetologia (2019) 62(10):1835–41. doi: 10.1007/s00125-019-4934-x PMC6731336 31451866

[180] Gao W Liu JL Lu X Yang Q. Epigenetic regulation of energy metabolism in obesity. J Mol Cell Biol (2021) 13(7):480–99. doi: 10.1093/jmcb/mjab043 PMC8530523 34289049

[181] Burgess S Smith GD Davie NM Dudbridge F Gill D Glymour MM. Guidelines for performing mendelian randomization investigations. version 2. Wellcome Open Res (2019) 4:186. doi: 10.12688/wellcomeopenres.15555.1 32760811 PMC7384151

[182] Burgess S Foley CN Allara E Staley JR Howson JMM. A robust and efficient method for mendelian randomization with hundreds of genetic variants. Nat Commun (2020) 11:376. doi: 10.1038/s41467-019-14156-4 31953392 PMC6969055

