# Corrigendum: The oxidative aging model integrated various risk factors in type 2 diabetes mellitus at system level

**DOI:** 10.3389/fendo.2024.1363078

**Published:** 2024-04-03

**Authors:** Yao Chen, Lilin Yao, Shuheng Zhao, Mengchu Xu, Siwei Ren, Lu Xie, Lei Liu, Yin Wang

**Affiliations:** ^1^ Department of Biomedical Engineering, School of Intelligent Medicine, China Medical University, Shenyang, Liaoning, China; ^2^ Shanghai-MOST Key Laboratory of Health and Disease Genomics & Institute for Genome and Bioinformatics, Shanghai Institute for Biomedical and Pharmaceutical Technologies, Shanghai, China; ^3^ Intelligent Medicine Institute, Fudan University, Shanghai, China; ^4^ Key Laboratory of GI Cancer Etiology and Prevention in Liaoning Province, The First Hospital of China Medical University, Shenyang, China

**Keywords:** oxidative stress, type 2 diabetes mellitus, energy metabolism, aging, pan-cancer analysis

In the published article, there was an error in **Figure 4** as published. The censored samples were missed in the previous version, herein they have been added. The corrected **Figure 4** and its caption appear below.

**Figure 4:**
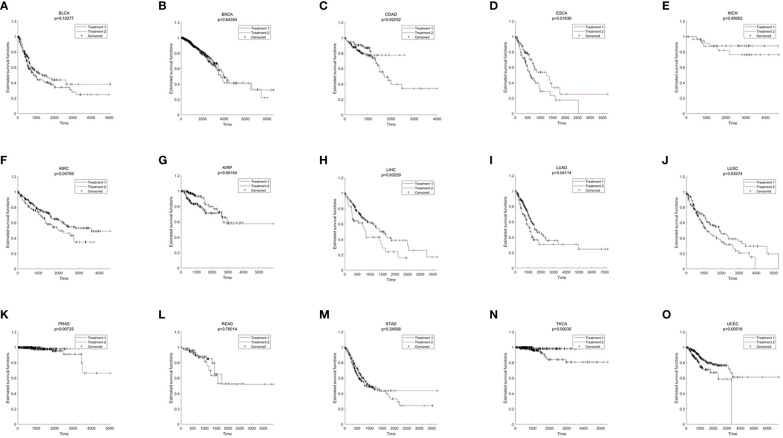
The results of survival analysis across different cancer types **(A)** BLCA; **(B)** BRCA; **(C)** COAD; **(D)** ESCA; **(E)** KICH; **(F)** KIRC; **(G)** KIRP; **(H)** LIHC; **(I)** LUAD; **(J)** LUSC; **(K)** PRAD; **(L)** READ; **(M)** STAD; **(N)** THCA; **(O)** LIHC.

In the published article, there was an error, which was related to **Figure 4**.

A correction has been made to **“Result 2.6”**, *Pan-cancer Analysis Further Verified the Mechanism of Oxidative aging in T2DM*, Paragraph 1. This sentence previously stated:

“There were 9 out of 15 cancer types with significant results (including BLCA, COAD, KIRC, KIRP, LUSC, PRAD, READ, THCA and UCEC, shown in **Figure 4**).”

The corrected sentence appears below:

“There were 9 out of 15 cancer types with significant results (including COAD, ESCA, KIRC, LIHC, LUAD, LUSC, PRAD, THCA and UCEC, shown in **Figure 4**).”

The authors apologize for this error and state that this does not change the scientific conclusions of the article in any way. The original article has been updated.

