# Supplementary figures and images for: The oxidative aging model integrated various risk factors in type 2 diabetes mellitus at system level
Source: Front Endocrinol (Lausanne). 2023 May 24;14:1196293. doi: 10.3389/fendo.2023.1196293 (PMC10244788; doi:10.3389/fendo.2023.1196293)

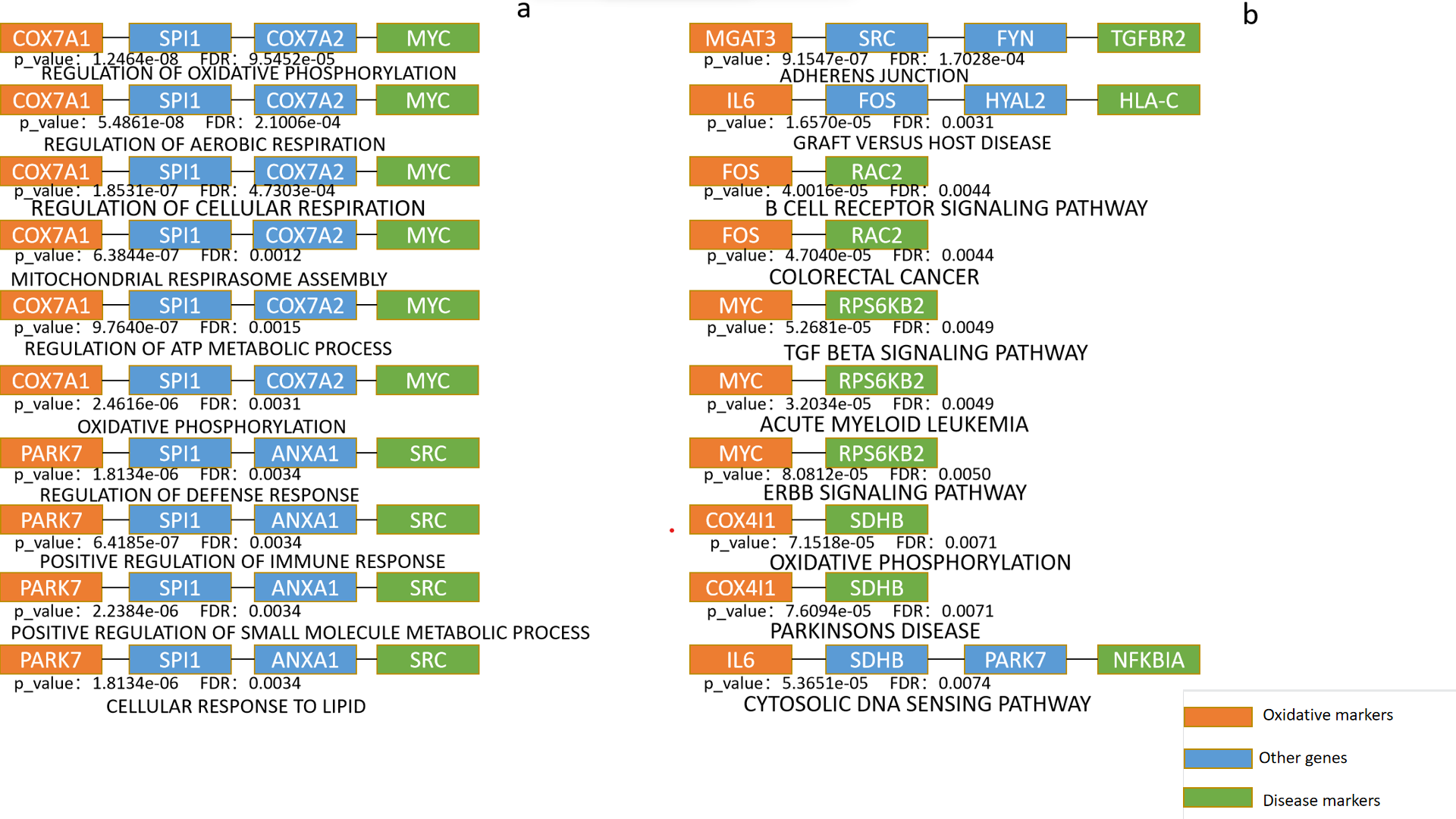

Supplement: Supplementary Table 1 — The detailed datasets used in this work. [file DataSheet_1.zip › Supplemental files/Figure S1.tif]

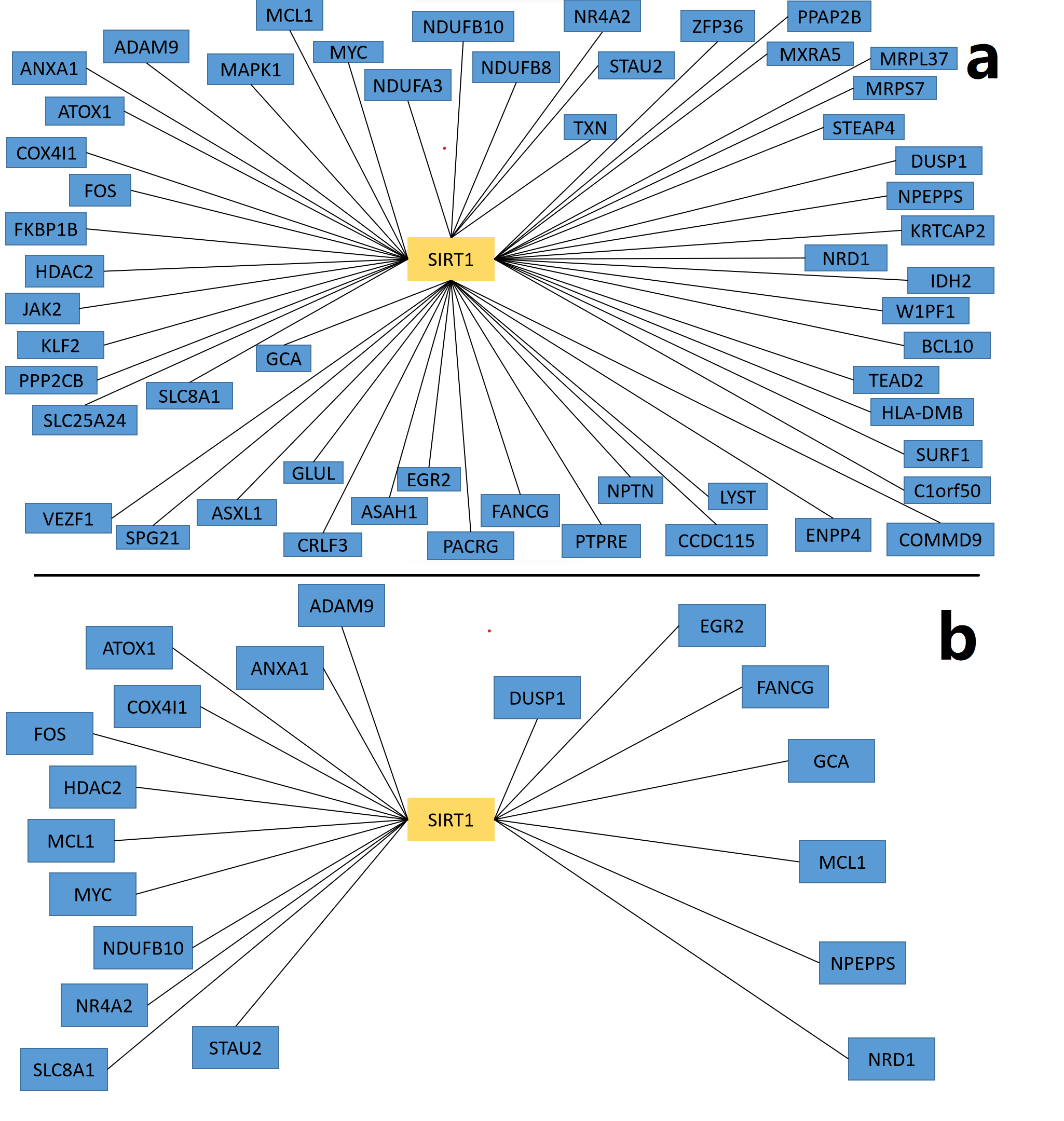

Supplement: Supplementary Table 1 — The detailed datasets used in this work. [file DataSheet_1.zip › Supplemental files/Figure S2.tif]

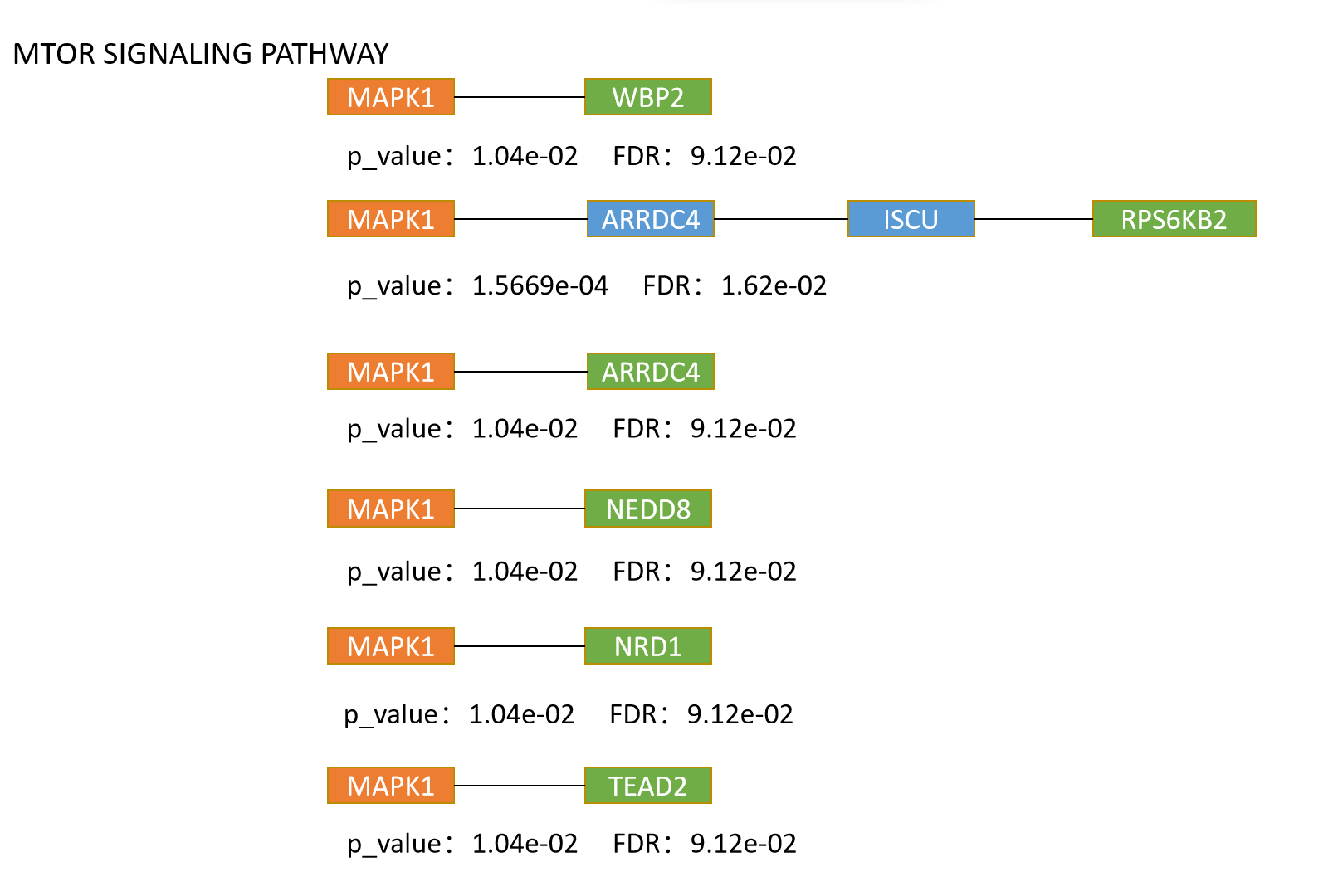

Supplement: Supplementary Table 1 — The detailed datasets used in this work. [file DataSheet_1.zip › Supplemental files/Figure S3.tif]

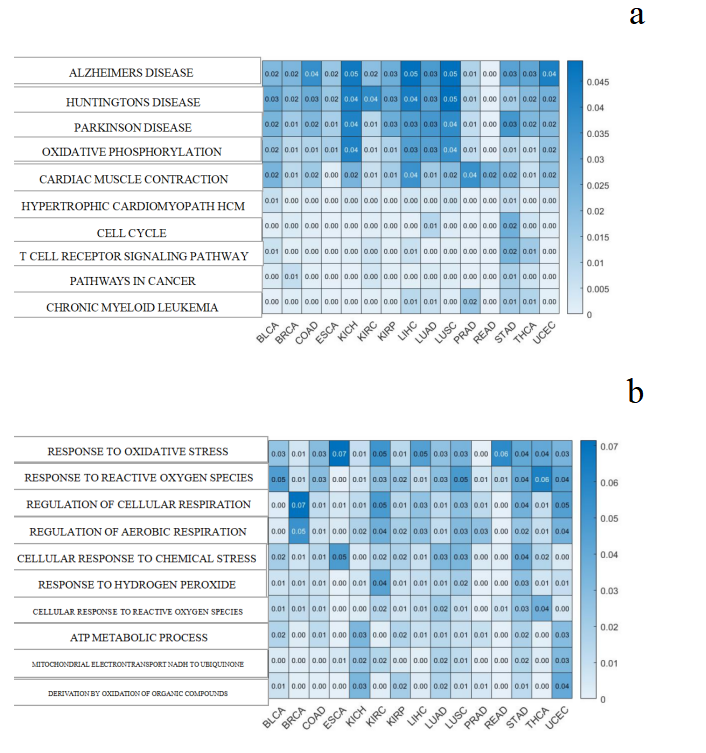

Supplement: Supplementary Table 1 — The detailed datasets used in this work. [file DataSheet_1.zip › Supplemental files/Figure S4.tif]
