# Supplementary material for: The oxidative aging model integrated various risk factors in type 2 diabetes mellitus at system level
Source: Front Endocrinol (Lausanne). 2023 May 24;14:1196293. doi: 10.3389/fendo.2023.1196293 (PMC10244788; doi:10.3389/fendo.2023.1196293)
Supplement: Supplementary Table 1 — The detailed datasets used in this work. [file DataSheet_1.zip › Supplemental files/Table S3-S7.doc]

Table S3 The data summarization in this work

|  | Training datasets | Test datasets | Total sample |
| --- | --- | --- | --- |
| Healthy young | 90 | 41 | 131 |
| Healthy aged | 145 | 63 | 208 |
| T2DM young | 25 | 15 | 40 |
| T2DM aged | 75 | 35 | 110 |

Table S4 the top 10 enriched KEGG pathways after sensitive analysis

| KEGG | Enriched shortest paths | Functions | Reference |
| --- | --- | --- | --- |
| PARKINSONS DISEASE | 109 | (1) T2DM and Parkinson Disease have shared pathological mechanism. (2) T2DM is a determinant of Parkinson Disease risk and progression. | Error: Reference source not found |
| CARDIAC MUSCLE CONTRACTION | 97 | Related to insulin sensitivity and mitochondrial function | Error: Reference source not found |
| HUNTINGTONS DISEASE | 95 | T2DM and Huntington’s Disease have shared treatment method. | Error: Reference source not found |
| OXIDATIVE PHOSPHORYLATION | 94 | Causing metabolic alterations at the organism level through producing energy-rich molecules like ATP. | Error: Reference source not found |
| ALZHEIMERS DISEASE | 94 | (1) T2DM is modifiable risk factor for Alzheimer’s Disease. (2) Insulin resistance is a common mechanism between Alzheimer’s Disease and T2DM. | Error: Reference source not found |
| TOLL LIKE RECEPTOR SIGNALING PATHWAY | 56 | Related to immune system. | Error: Reference source not found |
| COLORECTAL CANCER | 25 | T2DM is the risk factor for colorectal cancer. | Error: Reference source not found |
| ADHERENS JUNCTION | 22 | Regulating insulin vesicle trafficking. | Error: Reference source not found |
| MAPK SIGNALING PATHWAY | 16 | Inducing inflammation through activating environmental stress and cytokines. | Error: Reference source not found |
| PATHWAYS IN CANCER | 15 | T2DM is closely related to cancer. | Error: Reference source not found |

Table S5 The top 10 enriched BP terms after sensitive analysis

| BP | Enriched shortest paths | Functions | Reference |
| --- | --- | --- | --- |
| REGULATION OF AEROBIC RESPIRATION | 29 | (1) Regulating the level of glucose metabolism. (2) Reactive oxygen species (ROS) are a byproduct of aerobic respiration and signaling molecules, which controls various cellular functions. | Error: Reference source not found |
| CELLULAR RESPONSE TO CHEMICAL STRESS | 24 | Regulating the cellular redox state. | Error: Reference source not found |
| RESPONSE TO OXIDATIVE STRESS | 23 | (1) Causing the function of pancreatic beta cells damaged. (2) Related to insulin resistance. | Error: Reference source not found |
| REGULATION OF OXIDATIVE PHOSPH*ORYLATION | 21 | Playing role in energy production and free radical production. | Error: Reference source not found |
| RESPONSE TO REACTIVE OXYGEN SPECIES | 16 | (1) Modifying cell signaling proteins and then mediating T2DM. (2) As a central mechanism for the development of T2DM. | Error: Reference source not found |
| RESPONSE TO PLATELET DERIVED GROWTH FACTOR | 16 | Related to inflammation response. | Error: Reference source not found |
| RESPONSE TO LECTIN | 16 | Related to immune system. | Error: Reference source not found |
| MONOCYTE ACTIVATION | 14 | Important step in the normal immune response to pathogens and other related stimuli. | Error: Reference source not found |
| EPHRIN RECEPTOR SIGNALING PATHWAY | 14 | Related to inflammation response and cellular damage. | Error: Reference source not found |
| MITOCHONDRIAL RESPIRASOME ASSEMBLY | 14 | Regulating the production of energy. | Error: Reference source not found |

Table S6 KEGG pathways in each cancer with the minimum FDR after sensitive analysis

| Type of cancer | FDR | KEGG | Functions | Reference |
| --- | --- | --- | --- | --- |
| BLCA | 2.13e-04 | OXIDATIVE PHOSPHORYLATION | Cancer cells utilize certain pathways to enhance oxidative phosphorylation. | Error: Reference source not found |
| BLCA | 2.13e-04 | PARKINSONS DISEASE | Parkinson Disease and cancer share some common biological pathways, such as mitochondrial dysfunction and protein homeostasis. | Error: Reference source not found |
| BRCA | 7.82e-06 | INTESTINAL IMMUNE NETWORK FOR IGA PRODUCTION | Related to immune system. | Error: Reference source not found |
| COAD | 7.79e-06 | HUNTINGTONS DISEASE | Cancer and Huntington’s Disease have common pathogenesis. | Error: Reference source not found |
| ESCA | 8.857e-03 | CARDIAC MUSCLE CONTRACTION | Cardiac muscle contraction dysfunction is basic in cancer patients. | Error: Reference source not found |
| ESCA | 8.857e-03 | OXIDATIVE PHOSPHORYLATION | Cancer cells utilize certain pathways to enhance oxidative phosphorylation. | Error: Reference source not found |
| ESCA | 8.857e-03 | PARKINSONS DISEASE | Parkinson Disease and cancer share some common biological pathways, such as mitochondrial dysfunction and protein homeostasis. | Error: Reference source not found |
| KICH | 4.51e-05 | HUNTINGTONS DISEASE | Cancer and Huntington’s Disease have common pathogenesis. | Error: Reference source not found |
| KIRC | 1.09e-05 | PATHWAYS IN CANCER | Providing essential precursors for cancer cell growth, such as proteins, nucleic acids, and lipids. | Error: Reference source not found |
| KIRP | 4.32e-07 | ALZHEIMERS DISEASE | (1) age is the risk factor for the development of Alzheimer’s Disease and cancer. (2) some cancer patients may have a higher risk of Alzheimer’s Disease. | Error: Reference source not foundError: Reference source not found |
| LIHC | 2.5e-04 | ALZHEIMERS DISEASE | (1) age is the risk factor for the development of Alzheimer’s Disease and cancer. (2) some cancer patients may have a higher risk of Alzheimer’s Disease. | Error: Reference source not foundError: Reference source not found |
| LUAD | 4.51e-05 | HUNTINGTONS DISEASE | Cancer and Huntington’s Disease have common pathogenesis. | Error: Reference source not found |
| LUSC | 5.65e-06 | CARDIAC MUSCLE CONTRACTION | Cardiac muscle contraction dysfunction is basic in cancer patients. | Error: Reference source not found |
| PRAD | 1.0275e-02 | BLADDER CANCER | The ninth most common malignancy worldwide. | Error: Reference source not found |
| READ | 6.84e-03 | NEUROTROPHIN SIGNALING PATHWAY | Inhibiting cancer cells. | Error: Reference source not found |
| STAD | 4.73e-05 | HYPERTROPHIC CARDIOMYOPATHY HCM | Affecting protein homeostasis. | Error: Reference source not found |
| THCA | 3.44e-04 | HUNTINGTONS DISEASE | Cancer and Huntington’s Disease have common pathogenesis. | Error: Reference source not found |
| UCEC | 6.52e-06 | PARKINSONS DISEASE | Parkinson Disease and cancer share some common biological pathways, such as mitochondrial dysfunction and protein homeostasis. | Error: Reference source not found |

Table S7 BP terms of each cancer with the minimum FDR after sensitive analysis

| Type of cancer | FDR | BP | Functions | Reference |
| --- | --- | --- | --- | --- |
| BLCA | 2.98e-05 | REGULATION OF RESPONSE TO OXIDATIVE STRESS | Related to cancer, which can regulate the progression of cancer. | Error: Reference source not found |
| BRCA | 5.26e-07 | MONONUCLEAR CELL DIFFERENTIATION | Promoting macrophage differentiation and inducing inflammatory responses. | Error: Reference source not found |
| COAD | 1.53e-06 | RESPONSE TO INORGANIC SUBSTANCE | Increasing the risk of cancer, such as nitrate content. | Error: Reference source not found |
| ESCA | 2.18e-03 | REGULATION OF MITOCHONDRIAL ELECTRON TRANSPORT NADH TO UBIQUINONE | Cancer cell propagation is closely related to the regulation of the electron transport chain. | Error: Reference source not found |
| KICH | 5.26e-05 | ATP METABOLIC PROCESS | ATP is one of the main biochemical components of the tumor microenvironment. | Error: Reference source not found |
| KIRC | 2.42e-05 | RESPONSE TO IMMOBILIZATION STRESS | Enhancing the ability of some cancer cells to enter a dormant state. | Error: Reference source not found |
| KIRP | 1.59e-05 | ATP SYNTHESIS COUPLED ELECTRON TRANSPORT | Electron transport chain is required for cancer cell growth. | Error: Reference source not found |
| LIHC | 1.75e-04 | CELLULAR RESPONSE TO REACTIVE OXYGEN SPECIES | ROS dynamically affect the tumor microenvironment, and are known to initiate cancer angiogenesis, metastasis, and survival at various concentrations. | Error: Reference source not found |
| LUAD | 3.94e-07 | CELLULAR RESPONSE TO REACTIVE OXYGEN SPECIES | ROS dynamically affect the tumor microenvironment, and are known to initiate cancer angiogenesis, metastasis, and survival at various concentrations. | Error: Reference source not found |
| LUSC | 7.21e-05 | NEGATIVE REGULATION OF OXIDATIVE STRESS INDUCED NEURON DEATH | Neurons synapse directly to glioma cells and drive their proliferation and spread through glutamatergic action. | Error: Reference source not found |
| PRAD | 3.38e-04 | REGULATION OF OXIDATIVE PHOSPHORYLATION | Playing a crucial role in cancer progression. | Error: Reference source not found |
| READ | 1.105e-03 | RESPONSE TO OXIDATIVE STRESS | Related to cancer, which can regulate the progression of cancer. | Error: Reference source not found |
| STAD | 1.37e-05 | HEPATICOBILIARY SYSTEM DEVELOPMENT | Related to immune system and releasing inflammatory modulators. | Error: Reference source not found |
| THCA | 1.77e-05 | CELLULAR RESPONSE TO REACTIVE OXYGEN SPECIES | ROS dynamically affect the tumor microenvironment, and are known to initiate cancer angiogenesis, metastasis, and survival at various concentrations. | Error: Reference source not found |
| UCEC | 5.94e-06 | MITOCHONDRIAL RESPIRASOME ASSEMBLY | (1) Glycolysis is considered to be the major metabolic process in energy production and anabolic growth of cancer cells. (2) Mitochondrial respiratory defects may be the underlying cause of cancer. | Error: Reference source not foundError: Reference source not found |
